# Constitutive RLI Armoring Enhances CAR-NK Cell Effector Functions but Causes Lethal Toxicity In Vivo

**DOI:** 10.3390/ijms27083554

**Published:** 2026-04-16

**Authors:** Zhiming Ling, Yi Wang, Geping Wu, Wei Lin, Tao Lu, Guohua Yu, Jianxun Wang

**Affiliations:** School of Life Science, Beijing University of Chinese Medicine, Beijing 102488, China; lzm9527@bucm.edu.cn (Z.L.); 20230931204@bucm.edu.cn (Y.W.); bucm_linwei@163.com (W.L.);

**Keywords:** IL15, mbIL15, RLI, CAR-NK, B-cell malignancies, adoptive cell therapy, toxicity

## Abstract

Chimeric antigen receptor–natural killer (CAR-NK) cell therapy is a promising immunotherapy for hematological malignancies. While engineered interleukin15 (IL15) variants like membrane-bound IL15 (mbIL15) and the IL15/IL15Rα heterodimer (RLI) can enhance NK cell activity, their relative efficacy and safety as armor for CAR-NK cells remain unclear. This study systematically evaluated primary human CAR-NK cells co-expressing an anti-CD19 CAR (19ζ) with soluble IL15, mbIL15, or RLI. We found that 19ζ-RLI CAR-NK cells exhibited superior IL15 secretion, proliferation, cytotoxicity, and migration in vitro, and effectively controlled tumors in vivo. However, all IL15-armored constructs, particularly 19ζ-RLI, induced lethal toxicity in mice, characterized by CAR-NK hyperproliferation and elevated systemic IL15. Transcriptomic analysis revealed that this toxicity correlated with a hyperactive molecular state driven by persistent IL15 signaling. In conclusion, this study suggests that constitutive IL15 armoring can be a potent but risky strategy for enhancing CAR-NK cells, with RLI being the most potent yet toxic exemplar of this general principle. Our findings highlight the necessity of incorporating safety-optimized strategies, such as inducible cytokine expression, into the design of cytokine-armored CAR-NK therapies for clinical translation.

## 1. Introduction

CAR-NK cell therapy has emerged as another promising form of adoptive cell therapy, following the remarkable success of CAR-T therapy in hematologic malignancies [1,2]. NK cells possess innate anti-tumor activity, enabling them to eliminate malignant cells without requiring prior antigen priming [3]. NK cell recognition of tumors depends on the balance between activating and inhibitory signals, which allows NK cells to maintain anti-tumor activity even when tumor cells downregulate antigen expression to evade immune surveillance [4]. Recently, CAR-NK cells have demonstrated potent anti-tumor effects in both hematological malignancies and solid tumors [5,6]. Encouraging clinical outcomes regarding the efficacy and safety of CAR-NK cells have been reported, notably showing a low risk of cytokine release syndrome (CRS) and graft-versus-host disease (GVHD) [7,8].

Cytokine signaling has been demonstrated to play a crucial role in the proliferation, survival, and persistence of NK cells [9,10]. To overcome the inherently short lifespan of primary NK cells, most clinically advanced CAR-NK constructs are engineered to co-express interleukin15 (IL15). This strategy has demonstrated promising results, particularly in B-cell lymphoid malignancies [11,12]. One prominent approach involves the use of membrane-bound IL15 (mbIL15), created by fusing IL15 to the IL15 receptor alpha (IL15Rα) via a flexible linker [13,14,15]. Incorporating mbIL15 enhances NK cell functionality predominantly via cis-signaling, while its localized action improves safety by limiting systemic exposure [16]. Consequently, mbIL15-armored CAR-NK cells exhibit enhanced functionality and persistence through this autocrine mechanism, representing a promising therapeutic approach supported by recent studies [17,18].

Notably, the proteolytic cleavage of the ectodomain of full-length IL15Rα in the mbIL15 construct can release a soluble IL15/IL15Rα heterodimer (hetIL15) into the serum [19,20]. Based on this natural heterodimer, a recombinant cytokine known as RLI (IL15/IL15Rα sushi domain fusion complex) has been engineered [21]. RLI has been reported to possess potent immunostimulatory properties on human NK cells [22], promoting their proliferation and activation across multiple tumor preclinical models [23,24,25].

However, despite the well-documented efficacy of IL15 armoring, recent preclinical studies have raised concerns regarding its safety. Several independent investigations have demonstrated that constitutive expression of IL15 in CAR-NK cells can lead to severe systemic toxicity in immunodeficient mouse models, which was characterized by uncontrolled CAR-NK cell hyperproliferation, elevated serum IL15 levels, and fatal multi-organ damage [18,26,27]. Notably, such toxicity has been observed even in the absence of tumor stimulation and appears to be independent of cytokine release syndrome [10,27]. These findings underscore the critical need for careful safety evaluation of constitutive IL15 incorporation into CAR-NK designs and prompt a systematic comparison of the efficacy and safety profiles of different IL15-armored CAR-NK constructs, including RLI, which remains largely unexplored.

In this study, we aimed to generate three distinct IL15-armored CD19-targeting CAR-NK cell constructs (19ζ) derived from peripheral blood mononuclear cells (PBMCs): 19ζ-IL15, 19ζ-mbIL15, and 19ζ-RLI. We sought to systematically evaluate and compare their functional efficacy, persistence, and safety profiles through comprehensive in vitro and in vivo models. These comparative analyses are expected to elucidate the relative efficacy and safety profiles of these three IL15 formats in enhancing CAR-NK cell therapy.

## 2. Results

### 2.1. Generation of CD19-Specific CAR-NK Cells Armor with IL15, mbIL15 and RLI

**Figure 1:**
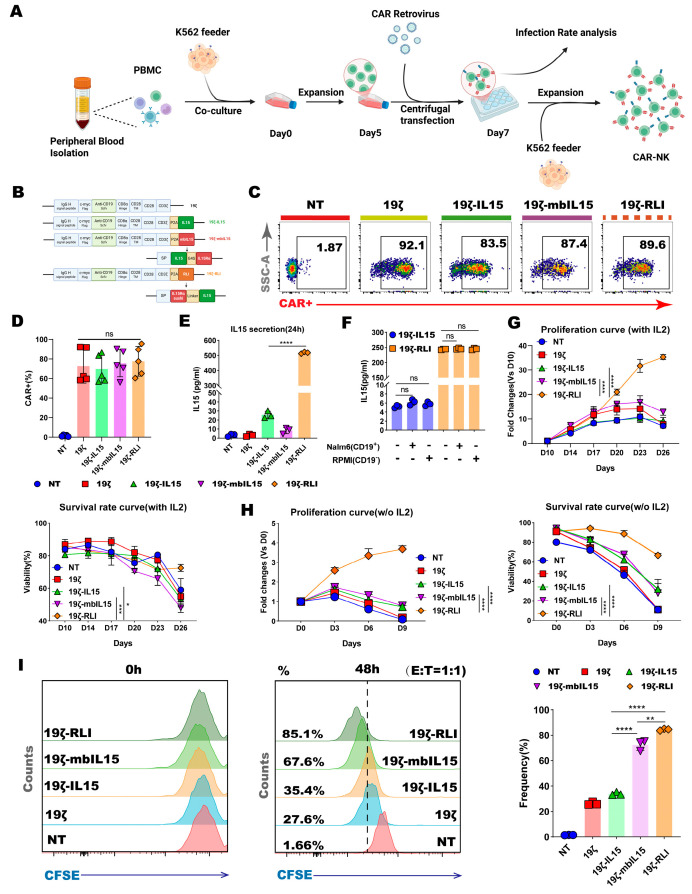
Generation, characterization, and proliferation of CAR-NK cells. (**A**) Schematic diagram showing the generation of CAR-NK cells from PBMC. (**B**) Schematic diagram of the 19ζ, 19ζ-IL15, 19ζ-mbIL15 and 19ζ-RLI constructs. (**C**) Representative flow cytometry plots showing CAR expression (Myc-tag) on transduced NK cells. (**D**) Quantification of CAR transduction efficiency from (**C**) (*n* = 5). (**E**) IL15 concentration in supernatant of NT, 19ζ, 19ζ-IL15, 19ζ-mbIL15, and 19ζ-RLI cells measured by ELISA (*n* = 3). (**F**) IL15 secretion from 19ζ-IL15 or 19ζ-RLI cells cultured alone or co-cultured with CD19^+^ Nalm6 or CD19^−^ RPMI cells (*n* = 3). (**G**,**H**) Proliferation and viability curves of the indicated NK cells cultured with IL2 (**G**) or without exogenous cytokines (**H**) (*n* = 3). (**I**) Proliferation of NK cells after co-culture with Raji cells was evaluated using CFSE staining (*n* = 3). Statistical comparisons were performed using one-way ANOVA with Tukey’s correction (**D**–**F**,**I**), two-way ANOVA with Tukey’s correction (**G**,**H**). Data are presented as the mean ± s.e.m. ns, no significant. * *p* < 0.05. ** *p* < 0.01. *** *p* < 0.001. **** *p* < 0.0001.

We first constructed a second-generation CD19-targeting CAR (designated 19ζ), which consisted of an anti-CD19 scFv, a CD8α hinge domain, a CD28 transmembrane and co-stimulatory domain, and a CD3ζ intracellular signaling domain. To generate cytokine-armored variants, sequences encoding three different forms of human IL15 were engineered: (1) soluble human IL15; (2) membrane-bound IL15 (mbIL15), created by fusing IL15 to the full-length IL15 receptor α chain (IL15Rα) via a (G4S)3 flexible linker; and (3) the recombinant cytokine RLI (IL15/IL15Rα sushi domain complex), constructed by fusing the sushi domain of IL15Rα to IL15 using a hinge and linker sequence. Each IL15 variant sequence was linked to the C-terminus of the 19ζ CAR via a P2A self-cleaving peptide, generating the final constructs: 19ζ-IL15, 19ζ-mbIL15, and 19ζ-RLI (Figure 1B).

Primary human NK cells were isolated and expanded from PBMC. These cells were then transduced using Baboon Envelope glycoprotein (BaEV)-pseudotyped retroviral vectors, as previously described (Figure 1A) [28]. Transduction efficiency, assessed by the expression of an N-terminal Myc-tag common to all CAR constructs, was comparable across the different NK cell groups (Figure 1C,D).

We next validated the expression and secretion profiles of the IL15 variants. ELISA of culture supernatants confirmed detectable IL15 secretion from 19ζ-IL15, 19ζ-mbIL15, and 19ζ-RLI cells (Figure 1E). As anticipated, supernatant from 19ζ-RLI cells contained substantially higher levels of IL15 (over 16-fold) compared to that from 19ζ-IL15 cells. The lowest but detectable IL15 in the 19ζ-mbIL15 supernatant is consistent with previously reported proteolytic cleavage of the membrane-tethered complex [20]. Furthermore, IL15 secretion from 19ζ-IL15 and 19ζ-RLI cells remained unchanged following co-culture with either CD19^+^ (Nalm6) or CD19^−^ (RPMI) tumor cells, indicating that secretion was constitutive and not influenced by antigen-specific stimulation (Figure 1F).

Phenotypic analysis of key NK cell surface markers revealed a similar distribution of major NK cell subsets across all engineered groups (Figure S2B,C). In summary, we successfully generated and validated a panel of CD19-specific CAR-NK cells armored with distinct forms of IL15.

### 2.2. RLI Is Superior to IL15 and mbIL15 in Promoting CAR-NK Cell Proliferation

We next evaluated the proliferative capacity of the distinct CAR-NK cells under various culture conditions. In the presence of recombinant human IL2 (rhIL2), all groups exhibited comparable proliferation and viability during the early culture phase. However, a clear divergence emerged at later time points. 19ζ-RLI cells uniquely sustained long-term proliferation after other groups had ceased expanding. Notably, 19ζ-RLI cells retained high viability even at day 26 from the initiation of culture. (Figure 1G).

To assess IL2 independent growth, we withdrew rhIL2 from the culture medium. This led to an immediate cessation of proliferation and a rapid decline in viability for both NT and 19ζ cells (Figure 1H). In contrast, 19ζ-IL15 and 19ζ-mbIL15 cells demonstrated a limited capacity for IL2-independent proliferation, initiating expansion at day 3 post-withdrawal. However, this proliferation was transient, and these cells subsequently underwent cell death, showing an overall rapid decrease in viability (Figure 1H). Notably, 19ζ-RLI cells continued to expand throughout the 9-day observation period in the absence of rhIL-2, and maintained viability above 80% until day 6 (Figure 1H).

We further investigated antigen-driven proliferation by co-culturing CFSE-labeled CAR-NK cells with CD19^+^ Raji cells. Baseline CFSE mean fluorescence intensity was similar across all groups at 0 h (Figure 1I). After 48, 72, and 96 h of co-culture, all IL15-armored CAR-NK cells displayed faster expansion compared to NT and 19ζ cells. Among them, 19ζ-RLI cells exhibited the most robust proliferation in response to tumor stimulation (Figure 1I and Figure S3A–D). It is worth noting that the CFSE dilution profiles appeared as a single peak rather than distinct multiple peaks, which is consistent with previous reports indicating that asymmetric cell division in lymphocytes can lead to atypical CFSE profiles [29,30]. In summary, these findings demonstrate that RLI armoring significantly enhances the proliferation and sustains the viability of CAR-NK cells.

### 2.3. RLI-Armored CAR-NK Cells Exhibit Superior Effector Functions In Vitro

**Figure 2:**
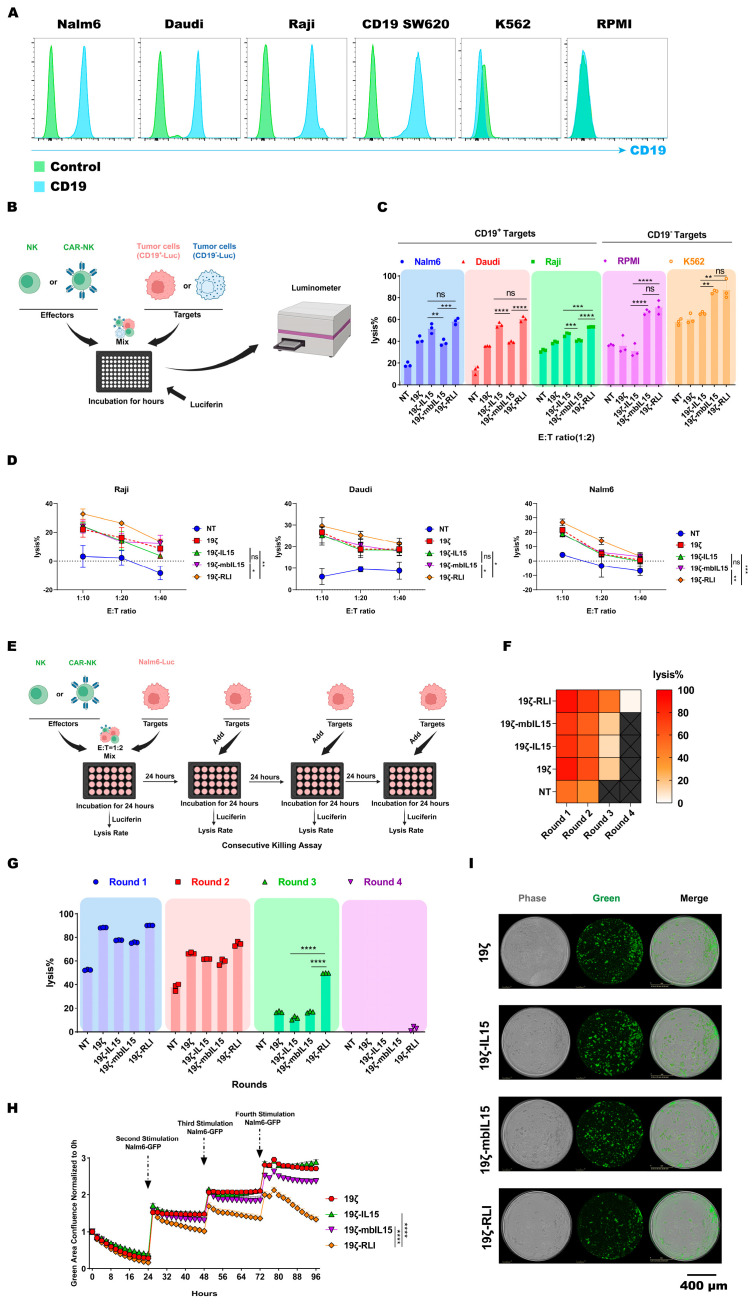
RLI enhances the cytotoxic function of CAR-NK cells. (**A**) Flow cytometry analysis of CD19 expression on Nalm6, Daudi, Raji, K562, RPMI, and CD19 SW620 cell lines. (**B**) Schematic of luciferase-based cytotoxicity assays. (**C**) Cytotoxicity of NT, 19ζ, 19ζ-IL15, 19ζ-mbIL15 and 19ζ-RLI cells against CD19^+^ target tumor cells or CD19^−^ target tumor cells at an E:T ratio of 1:2 (*n* = 3). (**D**) Cytotoxicity of the indicated NK cells against Raji, Daudi, and Nalm6 at the specified E:T ratios (1:10, 1:20, 1:40) (*n* = 3). (**E**) Schematic diagram of tumor-rechallenged luciferase-based cytotoxicity assays (*n* = 3). (**F**,**G**) Tumor rechallenge assay evaluating NT, 19ζ, 19ζ-IL15, 19ζ-mbIL15, and 19ζ-RLI cells against Nalm6 cells using luciferase-based readout (*n* = 3). (**H**) Tumor rechallenge assay of 19ζ, 19ζ-IL15, 19ζ-mbIL15, and 19ζ-RLI cells against Nalm6 cells monitored by IncuCyte live-cell imaging (*n* = 3). (**I**) Representative live-cell images at 96 h from the assay shown in (**H**). Statistical comparisons were performed using one-way ANOVA with Tukey’s correction (**C**,**G**) or two-way ANOVA with Tukey’s correction (**D**,**H**). Data are presented as the mean ± s.e.m. Scale bar, 400 μm. ns, no significant. * *p* < 0.05. ** *p* < 0.01. *** *p* < 0.001. **** *p* < 0.0001.

To evaluate the antigen-specific cytotoxic activity, engineered NK cells were co-cultured for 12 h with CD19^+^ (Raji, Daudi, Nalm6) or CD19^−^ (K562, RPMI) tumor cell lines at an E:T ratio of 1:2 (Figure 2A,B). As expected, 19ζ cells displayed significantly higher lysis of CD19^+^ targets compared to NT cells, while no difference was observed against CD19^−^ cell lines (Figure 2C). This confirms that the enhanced cytotoxicity is CAR-mediated and antigen-specific.

19ζ-RLI cells showed the highest cytotoxicity among all IL15-armored CAR-NK cells against the CD19^+^ targets tested, but statistically significant differences were observed only for Raji cells (Figure 2C), and the enhancement was modest. We reasoned that the supportive effect of cytokine signaling on NK cell function is cumulative and may require prolonged interaction to become fully apparent. Thus, we performed extended co-culture under a lower E:T ratio. Under these conditions, 19ζ-RLI cells demonstrated markedly enhanced cytotoxicity at an E:T ratios of 1:10, 1:20 and 1:40 (Figure 2D). We also assessed whether cytokine armoring influenced innate, antigen-independent cytotoxicity. When co-cultured with CD19-targets (K562 and RPMI), both 19ζ-mbIL15 and 19ζ-RLI cells exhibited stronger innate cytotoxicity compared to 19ζ-IL15 cells (Figure 2C).

To determine long-term functional persistence, we performed a multi-round tumor rechallenge assay. CAR-NK cells were repeatedly exposed to fresh Nalm6 cells every 24 h, and specific lysis was monitored using luciferase-based cytotoxicity assays and live-cell imaging (IncuCyte). Across four rounds of challenge, 19ζ-RLI cells maintained superior anti-tumor activity compared to both 19ζ-IL15 and 19ζ-mbIL15 cells (Figure 2E–H). This advantage became increasingly pronounced in later rounds (3 and 4). Intriguingly, live-cell imaging revealed that 19ζ-RLI cells continued to suppress tumor growth effectively even during the fourth rechallenge, whereas control by 19ζ-IL15 and 19ζ-mbIL15 cells was substantially diminished (Figure 2G,H). In summary, these functional analyses demonstrate that RLI armoring confers a superior capacity for sustained cytotoxicity.

### 2.4. RLI Enhances the Migratory Capacity of CAR-NK Cells

**Figure 3:**
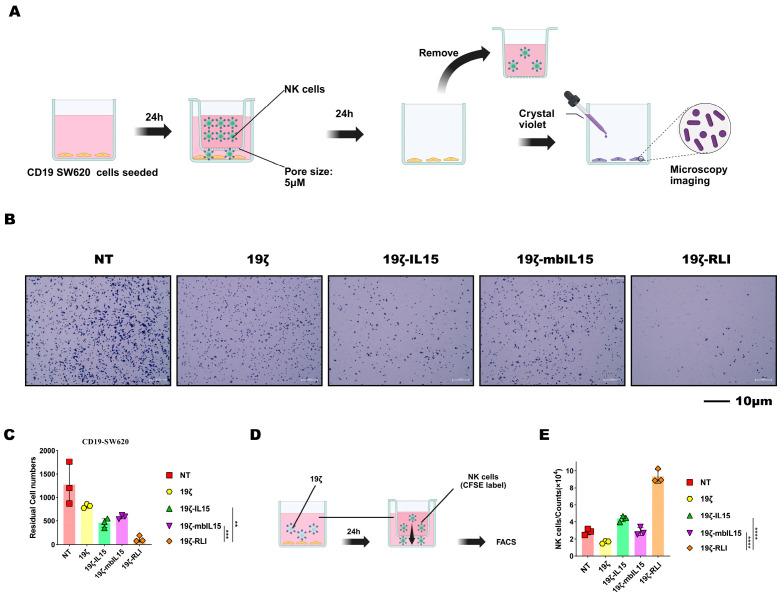
RLI enhances the migratory capacity of CAR-NK cells. (**A**) A schematic diagram of the migration cytotoxicity assay. (**B**) Representative microscopy images showing residual CD19 SW620 cells in the lower chamber after co-culture with the indicated NK cells. (**C**) Quantification of residual tumor cells from (**B**) (*n* = 3). (**D**) A schematic diagram of the migration capacity assay. (**E**) Quantification of CFSE-labeled NK cells that migrated to the lower chamber, analyzed by flow cytometry (*n* = 3). Statistical comparisons were performed using one-way ANOVA with Tukey’s correction (**C**,**E**). Data are presented as the mean ± s.e.m. Scale bar, 10 μm. ns, no significant. ** *p* < 0.01. *** *p* < 0.001. **** *p* < 0.0001.

Based on prior evidence that IL15 signaling regulates NK migration, we evaluated and compared the migratory capacity of CAR-NK cells via transwell migration assays [31,32,33]. In the first assay, CD19 SW620 cells were seeded in the lower chamber to serve as a target. After 24 h, NK cells were added to the upper chamber for an additional 24 h of co-culture. The residual tumor cells in the lower chamber were then quantified. Notably, the 19ζ-RLI group exhibited the fewest residual tumor cells, which could reflect superior migration cytotoxicity (Figure 3A,B).

To specifically quantify migration independently of cytotoxicity, a second transwell assay was designed. To eliminate the confounding effect of differential proliferation or activation induced by direct tumor contact, we used conditioned medium as the chemoattractant. This medium was collected from a 24 h co-culture of 19ζ CAR-NK cells and CD19 SW620 cells. CFSE-labeled NK cells were then seeded in the upper chamber (5 μm pore size) and allowed to migrate toward this conditioned medium for 24 h. Migrated CFSE^+^ cells in the lower chamber were counted by flow cytometry. This experiment revealed that 19ζ-RLI cells displayed a significantly higher number of migrated cells compared to 19ζ-IL15 and 19ζ-mbIL15 cells (Figure 3C,D). Taken together, findings from both transwell assays indicate that RLI-armored enhances the migratory capacity of CAR-NK cells, suggesting a potential for improved tumor infiltration in vivo.

### 2.5. RLI Enhanced CAR-NK Cell Degranulation and Cytotoxicity Cytokine Production

**Figure 4:**
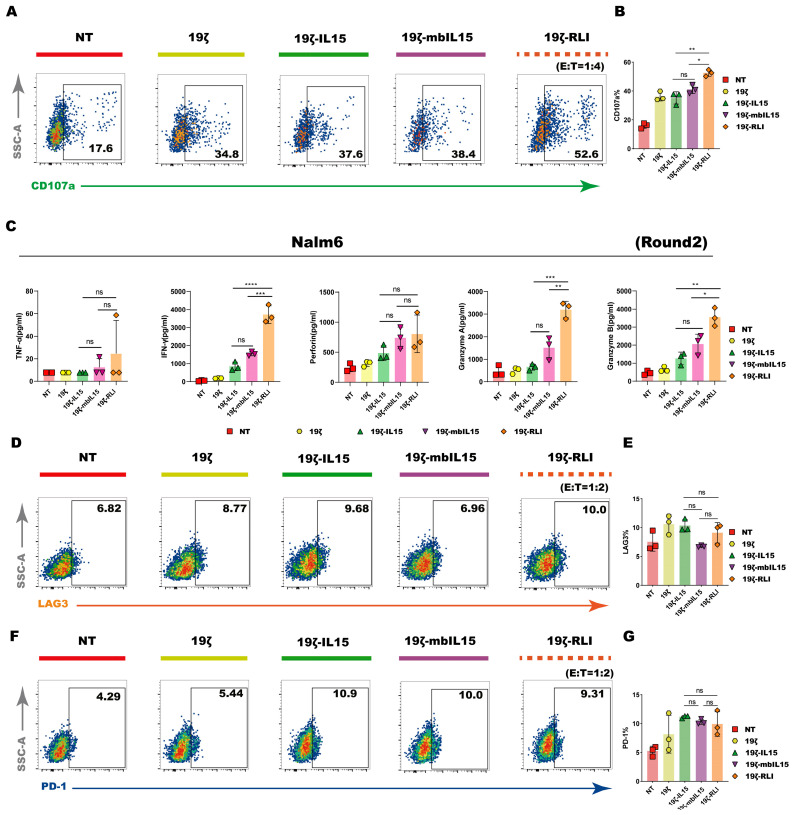
Degranulation and cytokine production of CAR-NK cells. (**A**) CAR-NK cells were co-cultured with Nalm6 cells at an E:T ratio of 1:4 for 4 h and subsequently harvested for analysis of the surface expression of CD107a. The representative flow cytometry plots were shown (*n* = 3). (**B**) Summary data of CD107a expression on NT, 19ζ, 19ζ-IL15, 19ζ-mbIL15 and 19ζ-RLI cells (*n* = 3). (**C**) Levels of TNFα, IFNγ, perforin, GZMA, and GZMB in the supernatant, measured by ELISA after two rounds of tumor rechallenge with Nalm6 cells (*n* = 3). (**D**–**G**) Representative flow cytometry plots showing the expression of exhaustion markers (PD-1 and LAG-3) on NT, 19ζ, 19ζ-IL15, 19ζ-mbIL15, and 19ζ-RLI cells after two rounds of tumor rechallenge with Nalm6 cells (*n* = 3). Statistical comparisons were performed using one-way ANOVA with Tukey’s correction (**B**,**C**,**E**,**G**). Data are presented as the mean ± s.e.m. ns, no significant. * *p* < 0.05. ** *p* < 0.01. *** *p* < 0.001. **** *p* < 0.0001.

To comprehensively evaluate the cytotoxic potential of CAR-NK cells, we analyzed both surface degranulation markers and cytokine production using flow cytometry and cytometric bead array (CBA). Prior short-term cytotoxicity assays showed minimal differences among IL15-armored CAR-NK cells at high E:T ratios. Given this, we evaluated CD107a expression following a 4 h co-culture at an E:T ratio of 1:1. All CAR-NK groups showed elevated CD107a compared to NT controls, yet no significant differences were detected among the three IL15 armed groups (Figure S4A). Since significant differences in cytotoxicity were observed at a lower E:T ratio in our earlier data, we repeated the degranulation assay at an E:T ratio of 1:4. In this setting, 19ζ-RLI cells displayed significantly higher CD107a expression than both 19ζ-IL15 and 19ζ-mbIL15 cells (Figure 4A,B).

Building on the observation that superior cytotoxicity of 19ζ-RLI cells emerged during repeated challenges, we evaluated cytokine release using a similar multi-round strategy. At an E:T of 1:2 ratio after a single round of stimulation, levels of TNFα, granzyme A (GZMA), granzyme B (GZMB) and perforin were comparable across all CAR-NK groups (Figure S4C). Notably, when cytokine production was examined under the second-round tumor rechallenge assay, the 19ζ-RLI group showed significantly higher secretion of IFNγ, GZMA, and GZMB compared to the other IL15 armed constructs, while 19ζ-IL15 and 19ζ-mbIL15 remained similar (Figure 4G).

Given the sustained cytotoxicity of 19ζ-RLI cells in repeated challenge assays, we further asked whether this enhanced function was accompanied by altered expression of the exhaustion markers PD-1, LAG-3, TIM3, and TIGIT. NK cells were co-cultured with Nalm6 at an E:T ratio of 1:2 and rechallenged with fresh tumor cells after 24 h. Expression of PD-1, LAG-3, TIM-3, and TIGIT was assessed, and no consistent reduction in any of these markers was observed in 19ζ-RLI cells compared to the other groups (Figure 4C–F and Figure S5). Taken together, these data demonstrate the functional superiority of 19ζ-RLI cells in degranulation and cytokine release under certain conditions.

### 2.6. RLI-Armored CAR-NK Displays Significant Control of Tumor Progression In Vivo but Induces Premature Mortality

**Figure 5:**
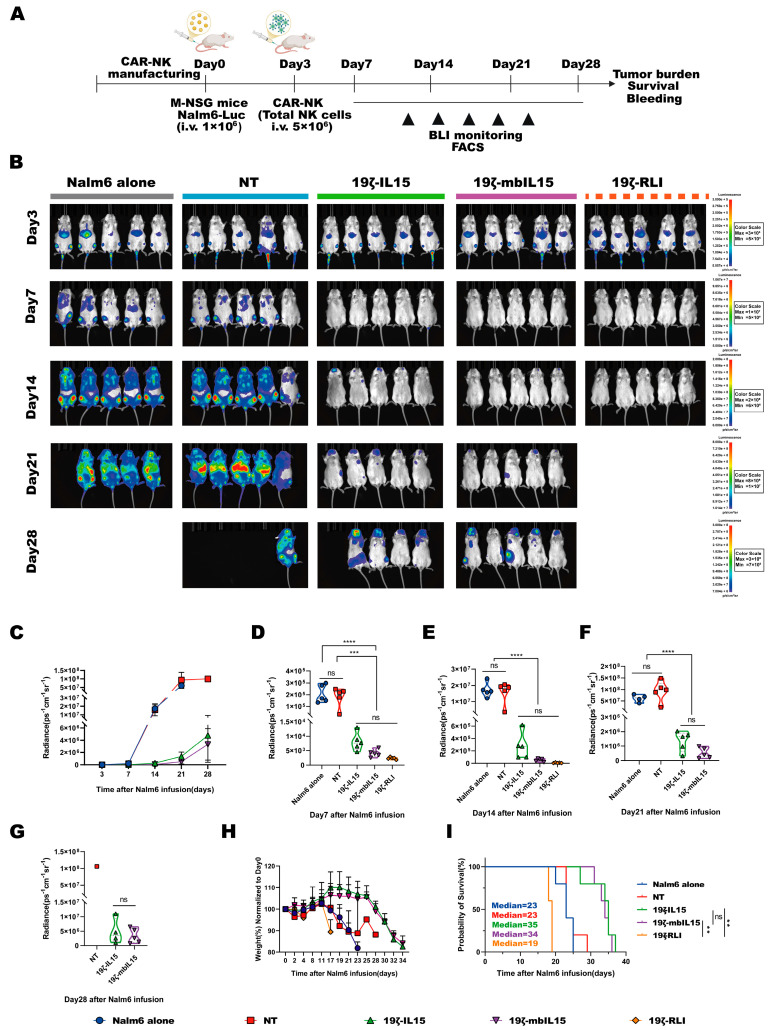
RLI-armored CAR-NK cells are associated with premature mortality in vivo. (**A**) Schematic of the experimental plan for the Nalm6 mouse model. (**B**–**G**) Tumor burden was monitored over time by bioluminescence imaging, with representative images (**B**) and quantitative data (**C**–**G**) shown (*n* = 5 mice per group). (**H**) Body weight changes in mice over the course of the experiment outlined in (**A**) (*n* = 5 mice per group). (**I**) Kaplan–Meier survival curves. Statistical comparisons were performed using one-way ANOVA with Tukey’s correction (**D**–**G**), logarithmic rank (Mantel–Cox) test (**I**). Data are presented as the mean ± s.e.m. ns, no significant. ** *p* < 0.01. *** *p* < 0.001. **** *p* < 0.0001.

To evaluate the anti-tumor efficacy and safety of distinct IL15-armored CAR-NK cells in vivo, we established a hematologic xenograft model. M-NSG mice were intravenously injected with 1 × 10^6^ Nalm6 cells expressing GFP and luciferase (Nalm6-GL). Three days later, the tumor burden was confirmed by in vivo imaging system (IVIS). The mice were then randomized into five groups and received a single dose intravenous injection of 5 × 10^6^ NT, 19ζ-IL15, 19ζ-mbIL15 or 19ζ-RLI cells, respectively (Figure 5A). All three IL15-armored CAR-NK cells mediated significant suppression of tumor progression compared to the untreated and NT cells. In contrast, tumor burden did not differ significantly between the two control groups (Nalm6 alone vs. NT), nor among the three IL15-armored CAR-NK groups themselves, as monitored by IVIS (Figure 5B–G).

Despite achieving a markedly low tumor burden, mice treated with 19ζ-RLI cells suffered rapid weight loss and premature death. Their median survival was shortened to 19 days, compared to 23 days for Nalm6 alone groups (*n* = 5; Figure 5I), indicating treatment-related lethal toxicity. In contrast, mice treated with 19ζ-IL15 or 19ζ-mbIL15 survived significantly longer than both NT and Nalm6 alone groups, with no difference in median survival between the two IL15-armored groups (19ζ-IL15: 35 days; 19ζ-mbIL15: 34 days; *n* = 5 per group; Figure 5I).

### 2.7. Hyperproliferation of IL15-Armored CAR-NK Cells Drives Lethal Toxicity In Vivo

**Figure 6:**
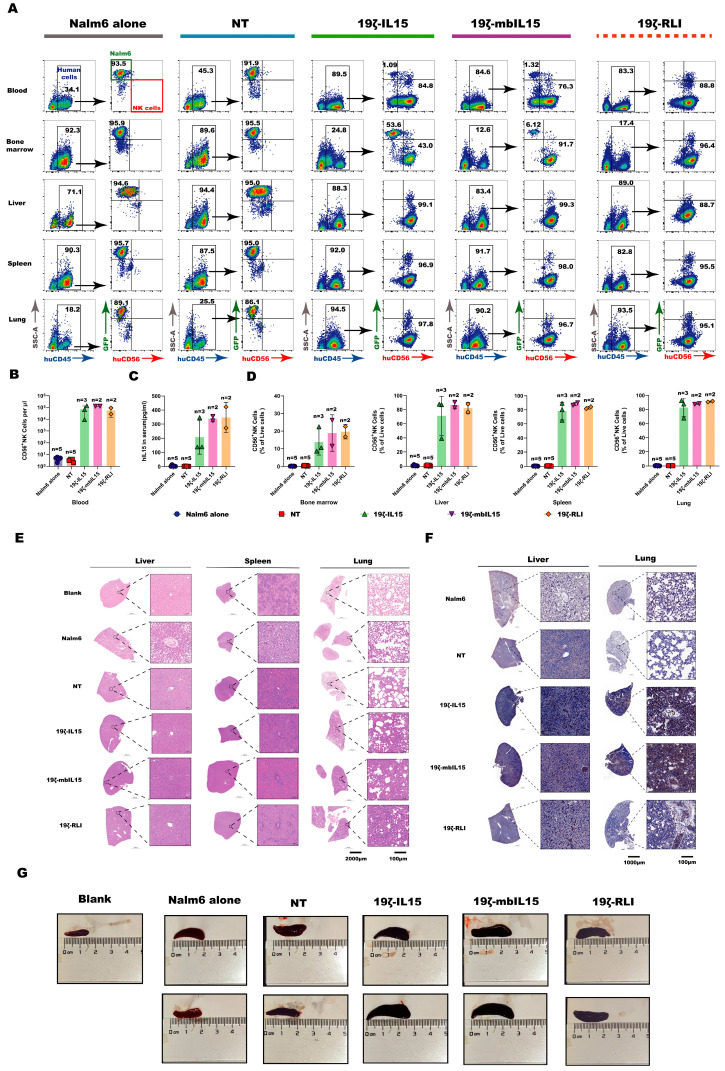
The hyperproliferation of IL15-armored CAR-NK cells led to lethal toxicity. (**A**) Peripheral blood, bone marrow, liver, spleen and lungs of mice were harvested at necropsy and analyzed by flow cytometry for the expression of hCD45, hCD56, and GFP. Representative flow cytometry plots were presented. (**B**–**D**) Quantification of human NK cells and IL15 levels at endpoint. (**B**) Human NK cells (Live^+^single^+^hCD45^+^hCD56^+^GFP^−^) in peripheral blood were quantified by flow cytometry. (**C**) Plasma concentrations of human IL15 were measured by ELISA. (**D**) Human NK cells in the indicated organs were quantified by flow cytometry. In panels (**B**–**D**), each data point represents one mouse, and the sample size (*n*) for each group is indicated above the corresponding bar. (**E**) Representative Hematoxylin and Eosin (H&E) staining images of the liver, spleen, and lungs from mice at necropsy. (**F**) Representative IHC CD56 staining images of liver and lung sections from mice at necropsy. (**G**) Photographs of the spleens of mice at necropsy. An age-matched, untreated mouse was included as a control (labeled “Blank”). Note on sample size: Mice were euthanized and tissues harvested after reaching predefined humane endpoints. Some mice in the treated groups succumbed before meeting these criteria, resulting in tissue autolysis and precluding analysis (these mice are not represented in the scatter plots). Consequently, the sample size (*n*) varies between groups and assays as indicated. Given the small and uneven sample sizes, data are presented descriptively without formal statistical comparison.

To investigate the mechanism underlying the lethal toxicity induced by 19ζ-RLI cells, we performed a comparative necropsy analysis of mice treated with 19ζ-RLI versus Nalm6 alone. We examined blood, bone marrow, liver, spleen, and lung for lymphoma and NK cell distribution (Figure S6). Notably, human NK cells were present at extremely high frequencies in peripheral blood, liver, spleen, and lung of mice treated with 19ζ-RLI cells. In contrast, Nalm6 alone mice showed high tumor burden but no detectable human NK cells in the same tissues (Figure 6A,B). These results indicate that 19ζ-RLI cells underwent marked hyperproliferation in vivo, which likely directly contributed to systemic toxicity and premature death.

Although mice treated with 19ζ-IL15 or 19ζ-mbIL15 cells exhibited extended survival compared to the Nalm6 alone groups, their tumor burden at the endpoint remained low. Thus, their eventual death was not primarily due to tumor progression. We therefore further asked whether hyperproliferation might be a common toxicity driver across all IL15-armored groups and extended the analysis accordingly. Results confirmed that 19ζ-IL15 and 19ζ-mbIL15-treated mice exhibited a similar pattern of NK cell hyperproliferation across multiple tissues, including peripheral blood, bone marrow, liver, spleen, and lungs (Figure 6A,B,D). Furthermore, high serum levels of human IL15 were detected in all IL15-armored groups at the endpoint (Figure 6C). To determine whether the observed toxicity was associated with cytokine release syndrome (CRS), we measured serum levels of mouse IL-6, IL-1β, and TNF-α, which were either below the limit of detection or present at extremely low levels in the majority of samples (Figure S7).

To corroborate these findings, liver, spleen, and lung tissues were collected for analysis. Compared with the blank control (age-matched, untreated mouse), all IL15-armored CAR-NK treatments resulted in abnormally enlarged spleens (Figure 6G), together with severe inflammatory infiltrates and substantial tissue damage in the liver, spleen, and lungs (Figure 6E). Critically, massive infiltration of human NK cells in the liver and lung was confirmed by immunohistochemical (IHC) staining, providing direct spatial evidence on CAR-NK cell hyperproliferation (Figure 6F). Collectively, these data supported that constitutive IL15 signaling drives uncontrolled CAR-NK cell expansion in vivo, accompanied by fulminant inflammatory tissue injury and lethal toxicity.

### 2.8. Transcriptomic Landscape Underlying Cytokine-Driven Hyperproliferation

**Figure 7:**
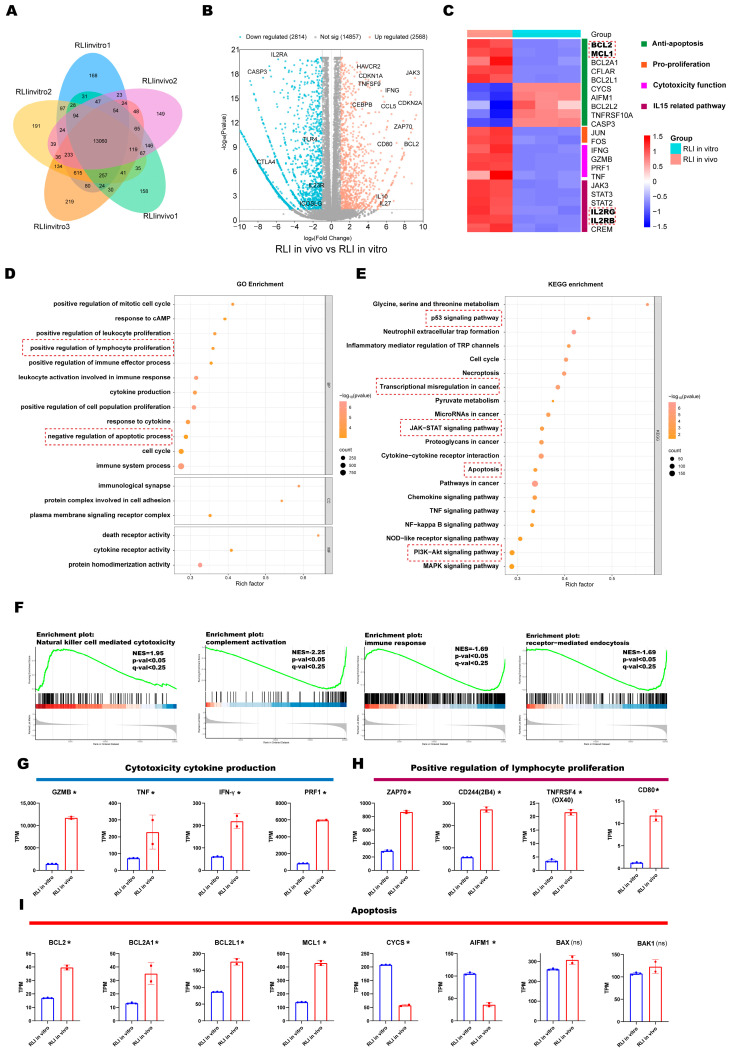
Transcriptomic landscape underlying cytokine-driven hyperproliferation. (**A**) Venn diagram depicting the shared and unique transcripts between in vivo-recovered and in vitro-cultured 19ζ-RLI cell. (**B**) Volcano plot of RNA-seq data showing differentially expressed genes (DEGs) in in vivo versus in vitro 19ζ-RLI cells (cut-offs: |log2FC| > 1, *p* < 0.05). (**C**) Comparative heatmap of key modules in in vivo versus in vitro 19ζ-RLI cells. (**D**,**E**) Bubble plots of enrichment terms from GO (**D**) and KEGG pathway (**E**) analyses of DEGs. Dot size represents gene count; color indicates *p*-value. Red dotted boxes in (C–E) highlight genes or pathways of primary relevance to this study. (**F**) GSEA plots showing enrichment of selected gene sets. (**G**–**I**) TPM (transcripts per million) values for genes associated with cytotoxicity and cytokine production (**G**), positive regulation of lymphocyte proliferation (**H**), and apoptosis regulation (**I**). Data are presented as the mean ± s.e.m. *, DEGs (|log2FC| > 1, *p* < 0.05); ns, non-DEGs.

Given that primary CAR-NK cells are generally reported to have limited in vivo persistence [34,35], and that 19ζ-RLI cells exhibited limited proliferative capacity when cultured in vitro without exogenous IL-2, the unexpected observation of their uncontrolled hyperproliferation and lethal toxicity in vivo prompted us to investigate the underlying mechanisms. To this end, we recovered 19ζ-RLI cells from recipient mice at necropsy and compared their transcriptional profiles to those of 19ζ-RLI cells maintained in vitro, aiming to define the transcriptomic signature associated with this hyperproliferative state. Therefore, we performed RNA-sequencing, using in vitro-cultured 19ζ-RLI cells as the baseline control. Unsupervised analysis of differentially expressed genes (DEGs) revealed a distinct transcriptomic signature in the in vivo-recovered cells (Figure 7A,B). A focused heatmap visualization (Figure 7C) of genes related to key modules (including apoptosis regulation, pro-survival signaling, cytotoxic effector function, and IL15 response) clearly delineates the two populations.

Further transcriptomic characterization revealed a molecular landscape consistent with cytokine-driven hyperproliferation and altered functionality. GO and KEGG enrichment analysis identified significant enrichment of pathways critically involved in cell survival and proliferation, including the apoptosis, JAK/STAT and PI3K/AKT signaling pathways, as well as the biological processes of negative regulation of apoptosis and positive regulation of lymphocyte proliferation (Figure 7D,E) [36,37]. Notably, we also observed enrichment in pathways associated with oncogenic states. These included transcriptional misregulation in cancer and the p53 signaling pathway, highlighting the dysregulated, cancer-like proliferation of these cells.

Mechanistically, the enrichment of IL15-related pathway, including IL15 receptor complex (Figure 7C), JAK/STAT and PI3K/AKT pathways, provided a molecular link to the elevated serum IL15 levels detected in vivo (Figure 7C and Figure S8A–C). This sustained signaling cascade likely drove the observed pro-survival and pro-proliferative phenotype. Concordantly, in vivo-recovered 19ζ-RLI cells exhibited a marked anti-apoptotic signature, with upregulation of genes such as *BCL2* and *MCL1* (Figure 7C,I) [38]. Furthermore, the positive regulation of lymphocyte proliferation module revealed elevated expression of key activation and co-stimulatory molecules (e.g., *ZAP70*, *2B4*, *OX40*, *CD80*), which collectively potentiate NK-cell expansion and activation (Figure 7H) [39,40].

Notably, the hyperproliferative state was accompanied by enhanced cytotoxic potential, as evidenced by GSEA enrichment for natural killer cell-mediated cytotoxicity (Figure 7F). Furthermore, this signature was driven by the pronounced upregulation of effector molecules such as *GZMB*, *IFNG*, *TNF*, and *PRF1* (Figure 7G). Finally, the hyperproliferative state was accompanied by broad functional impairments. There was a notable reduction in enrichment in gene sets related to immune response, receptor-mediated endocytosis, and complement activation (Figure 7F and Figure S8D). Taken together, these transcriptome alterations, driven by constitutive IL15 signaling, include higher survival, proliferation, and cytotoxicity, providing a reliable molecular explanation for the uncontrolled expansion.

## 3. Discussion

In this study, we generated CD19-targeted CAR-NK cells armored with three distinct IL15 variants. Among these, 19ζ-RLI cells exhibited the most potent in vitro effector functions but unexpectedly caused lethal hyperproliferation in vivo, characterized by uncontrolled expansion, elevated serum human IL15, and multi-organ damage. These findings reveal a critical safety risk associated with constitutive IL15 signaling in CAR-NK cells and underscore the need for CAR-NK designs that balance efficacy and safety when incorporating cytokine armoring strategies.

IL15 is critical for NK cell persistence, and incorporating IL15 signaling into CAR-NK designs is a promising strategy to improve in vivo efficacy [41]. To overcome the short half-life and potential toxicity of soluble IL15, engineered variants such as membrane-bound mbIL15 and the high-activity fusion RLI have been developed [23,42,43]. In our study, the 19ζ-RLI cells consistently showed the most potent activity across multiple in vitro assays, a finding that aligns with the reported superior bioactivity of the IL15/IL15Rα sushi domain fusion [23,24,25]. In addition, this enhanced functionality was not limited to antigen-specific killing. 19ζ-RLI cells also displayed stronger innate cytotoxicity against CD19-negative targets, suggesting that the RLI armoring CAR-NK may be particularly beneficial for treating tumors with heterogeneous antigen expression. Importantly, 19ζ-RLI cells mediated rapid and profound tumor regression, leading to undetectable tumor cells in all tissues examined in treated mice, which provides robust in vivo evidence of therapeutic efficacy. Moreover, a phase 1/1b trial (NCT04234113) on RLI demonstrated its potent pharmacological activity on T cells in cancer patients [22]. It is plausible that in immunocompetent settings, RLI-armored CAR-NK cells might further enhance anti-tumor immunity by activating endogenous T cells. Thus, RLI-armored CAR-NK cells represent a highly potent platform for cancer immunotherapy.

Despite this remarkable efficacy, the in vivo findings reveal a critical safety concern. All three IL15-armored CAR-NK cell groups induced robust hyperproliferation, leading to significant morbidity and organ damage. Notably, 19ζ-RLI cells caused the earliest mortality, which is consistent with their highest proliferative capacity. These observations are in line with recent preclinical reports showing that constitutive IL15 signaling in armored CAR-NK cells can drive lethal toxicity [10,18,27]. Consistent with this, serum mouse IL-6, IL-1β, and TNF-α levels were extremely low, arguing against a CRS-driven mechanism. Mechanistically, our transcriptome data defined a hyperproliferative state characterized by excessive IL15 signaling, anti-apoptotic and pro-survival shifts, and heightened cytotoxicity.

Furthermore, tumor recurrence coincided with CAR-NK hyperproliferation in the 19ζ-IL15 and 19ζ-mbIL15 groups. This seemingly paradoxical observation, that uncontrolled expansion occurred alongside tumor outgrowth, is rarely reported and offers a new perspective on the biology of IL15-armored CAR-NK cells. Our findings are consistent with prior reports that sustained IL15 signaling may impair NK cell functionality [44,45]. Notably, our transcriptomic data provide a molecular link to this paradox: the hyperproliferative state in 19ζ-RLI cells exhibited cancer-like transcriptional features, which may come at the cost of compromised normal immune function. Collectively, these observations suggest that constitutive IL15 expression may not be the optimal strategy for armoring CAR-NK cells, and that regulatable signaling approaches warrant further investigation.

Our results appear to contrast with a study reporting that mbIL15-armored CAR-NK cells had no observable toxicity, whereas soluble IL15-armored constructs proved lethal [18]. However, mbIL15-armored CAR-T cells have also been reported to induce lethal hyperproliferation in preclinical models [43,46]. In our study, elevated serum IL15 levels were also detected in the 19ζ-mbIL15 group. This phenomenon may be attributable to the hyperproliferation of these cells in vivo, accompanied by proteolytic shedding of the mbIL15 ectodomain [19,20]. Since mbIL15 primarily mediates autocrine stimulation, the high systemic IL15 levels likely reflect a consequence of the hyperproliferation [16]. Furthermore, the discrepancies across studies may reflect differences in specific mbIL15 constructs, dosing regimens, or model systems. Importantly, our work reveals a common risk of uncontrolled expansion driven by IL15-armored CAR-NK cells.

Several limitations should be acknowledged when interpreting these findings. First, the use of immunodeficient mice represents a key constraint, as a functional host immune system might restrain allogeneic cell expansion and mitigate toxicity [26]; future studies in humanized mouse models are warranted. Second, we did not longitudinally profile dynamic changes in serum IL15 levels or CAR-NK cell expansion in vivo; however, multi-organ flow cytometric analysis, H&E staining, and immunohistochemistry performed at experimental endpoints collectively yielded convergent evidence of CAR-NK hyperproliferation. Third, the use of a single dose makes it hard to exclude a contribution of cell dose to the observed toxicity. Notably, several preclinical studies have reported that reducing the cell dose of IL15-armored NK/CAR-NK cells did not prevent lethal hyperproliferation, as toxicity consistently occurred at a lower dose [10,18,27]. Moreover, a recent clinical case report described lethal hyperleukocytosis following IL15-armored CAR-NKT cell therapy. Remarkably, the initial NK cell purity of the infused product was extremely low, with astonishingly explosive expansion of CAR-NK cells, which contributed to fatal multiple organ failure [47]. Together, these preclinical and clinical findings raise the possibility that dose reduction alone may not fully eliminate the risk of such devastating toxicity, suggesting that regulatable IL15 expression systems or pharmacological safety switches may represent a more robust approach to ensuring safety [48,49].

## 4. Materials and Methods

### 4.1. Cell Lines and Culture Conditions

Human cancer cell lines of Raji, Nalm6, Daudi, K562, RPMI-8226(RPIMI), CD19 expressed SW620 (CD19 SW620) and 293T were obtained from American Type Culture Collection (ATCC). Raji, Nalm6, Daudi, K562, RPMI cells were cultured in RPMI-1640 basic medium (Gibco, Thermo Fisher Scientific, Waltham, MA, USA) with 10%FBS (Gibco) and 1% penicillin-streptomycin (Gibco). Baboon Envelope (BaEV)-293T and CD19 SW620 cells were cultured in DMEM medium (Gibco) supplemented with 10%FBS (Gibco) and 1% penicillin-streptomycin (Gibco). Cells were passaged every 2–3 days. Nalm6 and CD19 SW620 were transduced to express GFP to enable fluorescence monitoring by IncuCyte or detection by flow cytometry. Raji, Daudi, Nalm6, K562, RPMI cells were transduced to express firefly luciferase to enable NK cells cytotoxicity detection and bioluminescence in vivo imaging. All cells were maintained in 5% CO_2_ at 37 °C and were authenticated by STR profiling at the company of GENEWIZ (Tianjin, China). All cell lines were tested regularly for mycoplasma contamination using a Mycoplasma PCR Detection Kit (Beyotime, Shanghai, China) and were only used when tested negative for contamination.

### 4.2. PBMC-Derived NK Cells Generation and Expansion

Human PBMCs from healthy donors were used in this study. The study protocol received approval from the Ethics Committee of Beijing University of Chinese Medicine (Approval No. 2022BZYLL0102). PBMCs were isolated by density gradient centrifugation using Lymphoprep™ (Stem Cell Technologies, Vancouver, BC, Canada). To expand NK cells, K562 feeder cells were engineered to express 4-1BBL and membrane-bound IL21 (Figure S1A–C). Prior to co-culture, these feeder cells were treated with 50 µg/mL mitomycin C for 30 min to inhibit proliferation, followed by at least four washes with PBS. For NK cell expansion, isolated PBMCs were co-cultured with mitomycin C-treated feeder cells at a 1:1 ratio in NK medium (RPMI-1640 supplemented with 10% FBS and 1% penicillin/streptomycin (P/S)) containing 200 IU/mL recombinant human IL2 (rhIL2). The medium was refreshed every 2–3 days. Additional mitomycin C-treated feeder cells were added weekly to sustain NK cell expansion. Retroviral transduction of expanded NK cells was performed on day 5 post-stimulation (details described below). Cell counts and viability were regularly monitored using trypan blue staining (Invitrogen, Thermo Fisher Scientific, Waltham, MA, USA) and a Countess™ 2000 automated cell counter (Thermo Fisher Scientific, Waltham, MA, USA) to track proliferation and ensure equal cell numbers were used in subsequent functional assays.

### 4.3. Generation of Retroviral Constructs, Virus Production and CAR-NK Cells

The backbone CAR construct (designated 19ζ) comprised a humanized anti-human CD19 single-chain variable fragment (scFv), a CD8α hinge domain, a CD28 transmembrane and co-stimulatory domain, and a CD3ζ signaling domain. Sequences encoding human IL15 (NM_000585), membrane-bound IL15 (mbIL15; patent CN108728458A), or the heterodimeric IL15/IL15Rα complex (RLI; patent CN101360827A) were linked to the C-terminus of the 19ζ CAR via a P2A self-cleaving peptide. Each cassette was synthesized and subcloned into the pMFG retroviral vector, generating the final constructs: 19ζ-IL15, 19ζ-mbIL15, and 19ζ-RLI. Recombinant retroviruses were produced by transfecting these plasmids into 293T cells pseudotyped with the Baboon Envelope glycoprotein, as previously described [28]. Viral supernatants were collected 72 h post-transfection and stored at −80 °C until use. NK cells expanded for 5 days were transduced on RetroNectin (Takara, Kusatsu, Shiga, Japan, T100A)-coated plates according to the manufacturer’s protocol. Following retroviral transduction, the resulting cell products are termed 19ζ, 19ζ-IL15, 19ζ-mbIL15, and 19ζ-RLI NK cells. Non-transduced (NT) NK cells were used as controls. Transduction efficiency was assessed by flow cytometry 48–72 h later. Viral titers were determined on NK92MI cells using serial dilution.

### 4.4. ELISA Assay

To quantify constitutive IL15 secretion, 1 × 10^6^ CAR-NK cells were seeded per well of a 12-well plate in NK medium containing 200 IU/mL rhIL2. After 24 h, culture supernatants were collected by centrifugation and stored at −80 °C until analysis. To evaluate IL15 secretion in response to tumor stimulation, 4 × 10^4^ target cells (CD19^+^ or CD19^−^) were co-cultured with 2 × 10^4^ CAR-NK cells in a 96-well plate for 24 h. Supernatants were then collected and stored as above.

### 4.5. Luciferase-Based Cytotoxicity Assays

To assess cytotoxic activity, CD19^+^ (Raji, Daudi, Nalm6) or CD19^−^ (K562, RPMI) tumor cells were engineered to stably express firefly luciferase. NK cells (NT, 19ζ, 19ζ-IL15, 19ζ-mbIL15, or 19ζ-RLI) were co-cultured with target cells at an effector–target (E:T) ratio of 1:2 for 12 h. For dose–response evaluation, E:T ratios of 1:10, 1:20, and 1:40 were used in 24 h co-cultures. After incubation, Bright-Lumi™ Firefly Luciferase Reporter Gene Detection Reagent (Beyotime) was added, and luminescence was measured following a 5 min incubation using a SpectraMax i3x Multi-Mode Microplate Reader (Molecular Devices, San Jose, CA, USA).

For the tumor rechallenge assay, 4 × 10^4^ luciferase-expressing Nalm6 cells were co-cultured with 2 × 10^4^ NK cells in a 96-well plate. Every 24 h, fresh Nalm6 cells (4 × 10^4^ per well) were added to the same culture. Luminescence was measured before each rechallenge to quantify residual tumor cells, and specific lysis was calculated as described above.

### 4.6. Flow Cytometry

For flow cytometry analysis, cells were washed with PBS and centrifuged. The pellet was resuspended in FACS buffer (PBS containing 2% FBS) and incubated with the relevant antibody cocktail for 30 min at room temperature in the dark. Cells were then washed again and analyzed on a CytoFLEX flow cytometer (Beckman, Brea, CA, USA). Data were processed using FlowJo software (v10.8.2). CAR expression was detected using an APC-conjugated anti-c-Myc antibody (BioLegend, San Diego, CA, USA, 626810). The gating strategy for identifying NK cells is shown in Figure S2A.

For the carboxyfluorescein diacetate succinimidyl ester (CFSE) stain assay, NK cells were labeled with CFSE according to the manufacturer’s protocol. Briefly, 1 × 10^6^ cells were stained, and a sample was analyzed by flow cytometry to confirm uniform CFSE uptake. Then, 4 × 10^4^ CFSE-labeled NK cells were co-cultured with 4 × 10^4^ Raji cells in NK medium (without rhIL2) for 48 h, 72 h, or 96 h. Cells were harvested, stained with PE-Cy7 anti-human CD56 (BioLegend, 362510), and CFSE dilution within the CD56^+^ population was measured on the CytoFLEX cytometer.

The following antibodies were used for flow cytometry analysis: PE anti-human CD3 (Biolegend, 300308); PE-Cy7 anti-human CD56 (Biolegend, 362510); APC anti-c-myc (Biolegend, 626810); FITC anti-human CD16 (Biolegend, 302006); FITC anti-human CD107a (Biolegend, 328606); APC anti-human CD45 (Biolegend, 304012); APC anti-human LAG3 (Biolegend, 369212); APC anti-human PD-1 (Biolegend, 329908); APC anti-human CD19 (Biolegend, 302212); APC anti-human TIM3 (Biolegend, 345012); PE anti-human TIGIT (Biolegend, 372704). A complete list of antibodies used in this study is provided in Table S1. Data were acquired using CytoFLEX flow cytometer (Beckman), and analysis was performed using FlowJo software (v.10.8.2).

### 4.7. Degranulation Assay

NK cell degranulation was assessed by measuring surface CD107a expression. Briefly, 1 × 10^5^ or 4 × 10^5^ NK cells were co-cultured with 4 × 10^5^ Nalm6 target cells in NK medium (without rhIL2) in 24-well plates (Corning, NY, USA) for 4 h. Cells were then harvested, washed, and stained with PE anti-human CD3, PE-Cy7 anti-human CD56, and FITC anti-human CD107a antibodies. Surface CD107a expression on CD3-CD56^+^ NK cells were analyzed using a CytoFLEX flow cytometer.

### 4.8. Cytokine Release Assay

Cytokine production was evaluated using a two-round stimulation assay. In round 1, 2 × 10^4^ NK cells were co-cultured with 4 × 10^4^ Nalm6 cells in NK medium (without rhIL2) in a 96-well plate for 24 h. Half of the supernatant was collected and stored. For round 2, 2 × 10^4^ fresh Nalm6 cells were added to the same wells and cultured for an additional 24 h, after which the supernatant was collected. Cytokine concentrations in supernatants from both rounds were measured using the LEGENDplex™ Multi-Analyte Flow Assay Kit (Human CD8/NK Panel; BioLegend) according to the manufacturer’s instructions. Samples were acquired on a CytoFLEX flow cytometer, and data were analyzed with the LEGENDplex™ online analysis software (version 2025-05-01).

### 4.9. IncuCyte Live Cell Tumor Rechallenge

For real-time assessment of sustained cytotoxicity, 1 × 10^4^ NK cells were co-cultured with 2 × 10^4^ GFP-labeled Nalm6 cells in NK medium (without rhIL2) in a 96-well plate (Round 1). Every 24 h, half of the medium was gently removed and replaced with an equal volume of fresh NK medium containing 2 × 10^4^ GFP-labeled Nalm6 cells. This procedure was repeated for three additional rounds (Rounds 2–4). Throughout the assay, whole-well images were acquired in real time using an IncuCyte^®^ live-cell analysis system, and GFP-positive tumor cell counts were quantified using the integrated IncuCyte^®^ analysis software (version 2020c).

### 4.10. Transwell Assays

A transwell migration cytotoxicity assay was performed using 24-well transwell plates (5 µm pore; Beyotime, FTW034). First, 4 × 10^5^ CD19 SW620 cells were seeded in the lower chamber and incubated for 24 h. Then, 2 × 10^5^ NK cells were added to the upper chamber in NK medium (without rhIL2) and co-cultured for an additional 24 h at 37 °C. The upper chamber was removed, and cells in the lower chamber were washed with PBS, fixed with 4% paraformaldehyde for 10 min, and stained with crystal violet for 10 min. After washing and drying, images of five random fields per well were acquired using an Axiovert 5 microscope (ZEISS, Oberkochen, Germany), and residual tumor cells were quantified.

For the NK cell migration capacity assay, a conditioned medium-based transwell assay was employed. Conditioned medium was generated by co-culturing 4 × 10^5^ 19ζ CAR-NK cells with 4 × 10^5^ CD19 SW620 cells for 24 h. This supernatant was placed in the lower chamber of a transwell plate. CFSE-labeled NK cells (2 × 10^5^) were seeded in the upper chamber in NK medium (without rhIL2). After 24 h, cells that migrated to the lower chamber were collected and quantified by flow cytometry using counting beads (BioLegend). Results are expressed as the number of migrated CFSE^+^ NK cells.

### 4.11. Animal Experiment

All animal procedures were approved by the Institutional Animal Care and Use Committee of Beijing University of Chinese Medicine. Six- to eight-week-old female M-NSG mice (NOD.Cg-PrkdcscidIl2rgem1Smoc) were obtained from Shanghai Model Organisms Co., Ltd. (Shanghai, China). Mice were maintained in a sterile environment under a 12 h light/dark cycle at approximately 23 °C and 50% relative humidity, with ad libitum access to food and water. To establish a leukemia model, 1 × 10^6^ GFP and firefly luciferase-expressing Nalm6 cells were injected intravenously into each mouse on day 0. Three days later, mice with confirmed tumor engraftment (via bioluminescence imaging) were randomly assigned to treatment groups (5 mice per group): Nalm6 alone (tumor only), NT, 19ζ-IL15, 19ζ-mbIL15, or 19ζ-RLI, and received a single intravenous injection of 5 × 10^6^ NK cells (except the Nalm6 alone group). Tumor progression was monitored weekly by bioluminescence imaging using an AniView 100 multi-modal in vivo imaging system (BoLuTeng, Guangzhou, China) after intraperitoneal injection of D-luciferin (3 mg per mouse). Signal intensity was quantified as average radiance (p/s/cm^2^/sr) with AniView100 software (version 1.0.0).

At the endpoint, serum was obtained for human IL15, mIL1β, mIL6 and mTNF-α measurement using pre-coated ELISA kits. The following ELISA kits were used: human IL15 (Proteintech, Rosemont, IL, USA, KE00102), mIL1β (Proteintech, KE10003), mIL6 (Proteintech, KE10007), mTNF-α (Proteintech, KE10002). Liver, lung, spleen, and bone marrow were harvested for flow cytometric analysis of NK cell distribution or for histopathological examination after hematoxylin and eosin (H&E) staining. Mice were weighed every 2–5 days and monitored for signs of morbidity. Humane endpoints were defined as bilateral hind-limb paralysis or weight loss exceeding 20% of initial body weight, at which point mice were euthanized.

### 4.12. Immunohistochemical Staining

Formalin-fixed, paraffin-embedded tissue was routinely sectioned at 4 μm and placed on glass slides. After deparaffinization, anti-human CD56 (Proteintech, 60238) antibody was added to the tissue section to identify human NK cells. Twenty minutes of antigen retrieval in citrate buffer at 95 °C was performed prior to staining. Primary antibody was diluted at 1:100 with an incubation time of 60 min. Hematoxylin was used to counterstain the nuclei and sections were routinely cover-slipped. Slides were examined using a Leica DM2500 microscope (Leica Microsystems, Wetzlar, Germany) and images were captured with a Leica DMC6200 camera and SlideViewer software (version 2.5.0).

### 4.13. RNA-Sequence

Blood of mice was harvested at necropsy, and then 19ζ-RLI cells were sorted using MojoSort™ Human CD56 Nanobeads (Biolegend, 480156). RNA sequencing (RNA-seq) was performed in in vivo-recovered and in vitro-cultured 19ζ-RLI cells. Total RNA was extracted from the indicated NK cells. Library construction, sequencing, and quality control were conducted by Sangon Biotech Co., Ltd. (Shanghai, China). The raw RNA seq reading was compared with the human reference genome (hg38) using the Hisat2 v2.1.0 RNA seq comparative analysis tool. Differential expression analysis was performed using the DESeq2 R package (version 1.60.0). Genes with a log2 fold change value > 1 and an adjusted *p*-value < 0.05 are considered differentially expressed genes (DEGs). Gene Ontology (GO) enrichment analysis and Kyoto Encyclopedia of Genes and Genomes (KEGG) pathway analysis were performed using the cluster Profiler R package (version 4.14.4). Gene set enrichment analysis (GSEA) was performed using the local version of GSEA software (version 4.3.3). The GO and KEGG datasets were independently used for GSEA.

### 4.14. Western Blot

In vivo-recovered and in vitro-cultured 19ζ-RLI cells were lysed in RIPA buffer containing protease inhibitors on ice for 30 min. Protein concentrations were measured using a BCA kit (Beyotime, P0010S). Equal amounts of protein were separated by SDS-PAGE and transferred onto PVDF membranes. Membranes were blocked with 5% non-fat milk for 30 min and then incubated overnight with primary antibodies against STAT5 (CST, 25656), p-STAT5 (CST, 4322), and β-actin (Proteintech, 66009-1-Ig). After washing with TBST, membranes were incubated with HRP-conjugated secondary antibodies (Proteintech, SA00001-2) for 60 min. Signals were detected using an enhanced chemiluminescence (ECL) substrate.

### 4.15. Statistical Analysis

All statistical analyses were performed using GraphPad Prism software (version 8.02). Data are presented as mean ± s.e.m. unless otherwise noted. For comparisons among three or more groups, one-way or two-way ANOVA with appropriate post hoc tests was applied. Survival curves were generated using the Kaplan–Meier method and compared with the log-rank (Mantel–Cox) test. Statistical significance was defined as *p* < 0.05. The specific statistical test used and the sample size (*n*) for each experiment are indicated in the corresponding figure legends. Significance levels are denoted as follows: ns, not significant; * *p* < 0.05; ** *p* < 0.01; *** *p* < 0.001; **** *p* < 0.0001.

## 5. Conclusions

Our study demonstrates that RLI-armored CAR-NK cells exhibit superior proliferation, anti-tumor activity, and persistence in vitro. More importantly, our in vivo data reveal a previously underappreciated risk that IL15-armored CAR-NK cells can induce lethal toxicity in xenograft models through cytokine-driven hyperproliferation. These findings underscore the critical importance of safety evaluation for IL15-armored therapies prior to clinical translation. Moving forward, it is imperative to explore safer engineering strategies. Examples include inducible cytokine expression and integrated safety switches. These innovations are essential to harness the therapeutic potential of IL15 while preventing uncontrolled proliferation.

## Data Availability

The original contributions presented in this study are included in the article. Further inquiries can be directed to the corresponding author.

## Supplementary Materials

The following supporting information can be downloaded at: https://www.mdpi.com/article/10.3390/ijms27083554/s1.

## Abbreviations

The following abbreviations are used in this manuscript:

NK	Natural killer
CAR	Chimeric antigen receptor
IL15	Interleukin15
mbIL15	membrane-bound IL15
RLI	receptor linker IL15
CRS	cytokine release syndrome
GVHD	graft-versus-host disease
IL15Rα	IL15 receptor alpha
hetIL15	IL15/IL15Rα heterodimer
PBMCs	peripheral blood mononuclear cells
ATCC	American Type Culture Collection
ScFv	single-chain variable fragment
NT	Non-transduced
BaEV	Baboon Envelope
RNA-seq	RNA sequencing
E:T ratio	effector: target ratio
CBA	cytometric bead array
IVIS	in vivo imaging system
H&E	Hematoxylin and Eosin
IHC	immunohistochemical
GZMA	granzyme A
GZMB	granzyme B

## References

[1] Peng L., Sferruzza G., Yang L., Zhou L., Chen S. (2024). CAR-t and CAR-NK as cellular cancer immunotherapy for solid tumors. Cell. Mol. Immunol..

[2] Yazdanparast S., Bakhtiyaridovvombaygi M., Davoodi-Moghaddam Z., Asadi G., Monjezi F., Kiyamehr P., Gharehbaghian A., Abroun S., Moradi N. (2025). CAR-NK cell therapy in multiple myeloma: From preclinical and clinical landscape to joining the force for treatment strategies optimization. Cell Commun. Signal..

[3] Kyrysyuk O., Wucherpfennig K.W. (2023). Designing cancer immunotherapies that engage t cells and NK cells. Annu. Rev. Immunol..

[4] Vivier E., Rebuffet L., Narni-Mancinelli E., Cornen S.P., Igarashi R.Y., Fantin V.R. (2024). Natural killer cell therapies. Nature.

[5] Bahramloo M., Shahabi S.A., Kalarestaghi H., Rafat A., Mazloumi Z., Samimifar A., Asl K.D. (2024). CAR-NK cell therapy in AML: Current treatment, challenges, and advantage. Biomed. Pharmacother..

[6] Luo J., Guo M., Huang M., Liu Y., Qian Y., Liu Q., Cao X. (2025). Neoleukin-2/15-armored CAR-NK cells sustain superior therapeutic efficacy in solid tumors via c-myc/NRF1 activation. Signal Transduct. Target. Ther..

[7] Lei W., Liu H., Deng W., Chen W., Liang Y., Gao W., Yuan X., Guo S., Li P., Wang J. (2025). Safety and feasibility of 4-1BB co-stimulated CD19-specific CAR-NK cell therapy in refractory/relapsed large b cell lymphoma: A phase 1 trial. Nat. Cancer.

[8] Marin D., Li Y., Basar R., Rafei H., Daher M., Dou J., Mohanty V., Dede M., Nieto Y., Uprety N. (2024). Safety, efficacy and determinants of response of allogeneic CD19-specific CAR-NK cells in CD19(+) b cell tumors: A phase 1/2 trial. Nat. Med..

[9] Ma S., Caligiuri M.A., Yu J. (2022). Harnessing IL-15 signaling to potentiate NK cell-mediated cancer immunotherapy. Trends Immunol..

[10] Shanley M., Daher M., Dou J., Li S., Basar R., Rafei H., Dede M., Gumin J., Pantaleόn Garcίa J., Nunez Cortes A.K. (2024). Interleukin-21 engineering enhances NK cell activity against glioblastoma via CEBPD. Cancer Cell.

[11] Liu E., Marin D., Banerjee P., Macapinlac H.A., Thompson P., Basar R., Nassif Kerbauy L., Overman B., Thall P., Kaplan M. (2020). Use of CAR-transduced natural killer cells in CD19-positive lymphoid tumors. N. Engl. J. Med..

[12] Lin P., Acharya S., Silva F.R., Basar R., Uprety N., Rueda L.Y.M., Lin P., Gilbert A.L., Banerjee P.P., Fang D. (2025). CD70-targeting CAR-NK cells overcome BCMA downregulation and improve survival in high-risk multiple myeloma models. Blood Cancer Discov..

[13] Burga R.A., Aksoy B.A., Ao Z., Tchaicha J.H., Sethi D.K., Villasmil Ocando A., Kulkarni G.S., Lajoie S., Pedro K.D., Tremblay J.R. (2025). IL-2-independent expansion, persistence, and antitumor activity in TIL expressing regulatable membrane-bound IL-15. Mol. Ther. J. Am. Soc. Gene Ther..

[14] Mortier E., Woo T., Advincula R., Gozalo S., Ma A. (2008). IL-15ralpha chaperones IL-15 to stable dendritic cell membrane complexes that activate NK cells via trans presentation. J. Exp. Med..

[15] Webb L., Lofgren M., Patterson T., Watt A., Lajoie J., Zieba A., Fleury M., Liu E., Ding J., Tighe R. (2025). Membrane-bound IL-15 co-expression powers a potent and persistent CD70-targeted TRuC t-cell therapy. Front. Immunol..

[16] Imamura M., Shook D., Kamiya T., Shimasaki N., Chai S.M.H., Coustan-Smith E., Imai C., Campana D. (2014). Autonomous growth and increased cytotoxicity of natural killer cells expressing membrane-bound interleukin-15. Blood.

[17] Feng D., Sun L., Hu D., Wu J., Xu F., Kong X., Yang R., Jiang G., Jiang L., Wang S. (2025). Expression of membrane-bound interleukin-15 sustains the growth and survival of CAR-NK cells. Int. Immunopharmacol..

[18] Xu X., Cao P., Wang M., Wan Y., Sun S., Chen Y., Liu Y., Su T., Gao G., Liu X. (2025). Signaling intact membrane-bound IL-15 enables potent anti-tumor activity and safety of CAR-NK cells. Front. Immunol..

[19] Bergamaschi C., Bear J., Rosati M., Beach R.K., Alicea C., Sowder R., Chertova E., Rosenberg S.A., Felber B.K., Pavlakis G.N. (2012). Circulating IL-15 exists as heterodimeric complex with soluble IL-15rα in human and mouse serum. Blood.

[20] Mortier E., Bernard J., Plet A., Jacques Y. (2004). Natural, Proteolytic Release of a Soluble Form of Human IL-15 Receptor α-Chain That Behaves as a Specific, High Affinity IL-15 Antagonist. J. Immunol..

[21] Bouchaud G.G., Garrigue-Antar L., Solé V.R., Quéméner A.S., Boublik Y., Mortier E., Perdreau H., Jacques Y., Plet A. (2008). The exon-3-encoded domain of IL-15ralpha contributes to IL-15 high-affinity binding and is crucial for the IL-15 antagonistic effect of soluble IL-15ralpha. J. Mol. Biol..

[22] Champiat S., Garralda E., Galvao V., Cassier P.A., Gomez-Roca C., Korakis I., Grell P., Naing A., LoRusso P., Mikyskova R. (2025). Nanrilkefusp alfa (SOT101), an IL-15 receptor βγ superagonist, as a single agent or with anti-PD-1 in patients with advanced cancers. Cell Rep. Med..

[23] Bessard A., Solé V.R., Bouchaud G.G., Quéméner A.S., Jacques Y. (2009). High antitumor activity of RLI, an interleukin-15 (IL-15)-IL-15 receptor alpha fusion protein, in metastatic melanoma and colorectal cancer. Mol. Cancer Ther..

[24] Desbois M.L.N., Béal C., Charrier M.L.D., Besse B., Meurice G., Cagnard N., Jacques Y., Béchard D., Cassard L., Chaput N. (2020). IL-15 superagonist RLI has potent immunostimulatory properties on NK cells: Implications for antimetastatic treatment. J. Immunother. Cancer.

[25] Vincent M., Teppaz G.R., Lajoie L., Solé V.R., Bessard A., Maillasson M., Loisel S., Béchard D., Clémenceau B., Thibault G. (2014). Highly potent anti-CD20-RLI immunocytokine targeting established human b lymphoma in SCID mouse. mAbs.

[26] Baughan S.L., Folsom T., Johnson M., Kreuger J., Webber B., Moriarity B.S. (2025). Perspective: IL-15 cytokine-armored NK cells as ready-to-use immunotherapy for diverse malignancies: Therapeutic potential and toxicity risks. Front. Immunol..

[27] Christodoulou I., Ho W.J., Marple A., Ravich J.W., Tam A., Rahnama R., Fearnow A., Rietberg C., Yanik S., Solomou E.E. (2021). Engineering CAR-NK cells to secrete IL-15 sustains their anti-AML functionality but is associated with systemic toxicities. J. Immunother. Cancer.

[28] Zhao L., Qiu C., Chen H., Yu Z., Fan J., Ma Q., Zhan S., Feng Y., Li X., Ma P. (2025). Construction of stable packaging cell lines for large-scale industrial BaEV-enveloped retroviral vector production. Front. Immunol..

[29] Xia Y., Zhu J., Guo R., Shen X., Shi M., Shen N., Fan L., Chen L. (2025). Preclinical evaluation of TIGIT as a target to enhance efficacy and mitigate T cell exhaustion in multiple myeloma following BCMA-CAR-T therapy. Cell Death Dis..

[30] Bocharov G., Luzyanina T., Cupovic J., Ludewig B. (2013). Asymmetry of cell division in CFSE-based lymphocyte proliferation analysis. Front. Immunol..

[31] Bergamaschi C., Pandit H., Nagy B.A., Stellas D., Jensen S.M., Bear J., Cam M., Valentin A., Fox B.A., Felber B.K. (2020). Heterodimeric IL-15 delays tumor growth and promotes intratumoral CTL and dendritic cell accumulation by a cytokine network involving XCL1, IFN-γ, CXCL9 and CXCL10. J. Immunother. Cancer.

[32] Oner A., Kobold S. (2022). Transwell migration assay to interrogate human CAR-t cell chemotaxis. STAR Protoc..

[33] Yao X., Matosevic S. (2021). Chemokine networks modulating natural killer cell trafficking to solid tumors. Cytokine Growth Factor Rev..

[34] Rafei H., Basar R., Acharya S., Hsu Y.S., Liu P., Zhang D., Bohn T., Liang Q., Mohanty V., Upadhyay R. (2025). CREM is a regulatory checkpoint of CAR and IL-15 signalling in NK cells. Nature.

[35] Lin Y., Xiao Z., Hu F., Zheng X., Zhang C., Wang Y., Liu Y., Huang D., Wang Z., Xia C. (2025). Engineered CRO-CD7 CAR-NK cells derived from pluripotent stem cells avoid fratricide and efficiently suppress human t-cell malignancies. J. Hematol. Oncol..

[36] Bottos A., Gotthardt D., Gill J.W., Gattelli A., Frei A., Tzankov A., Sexl V., Wodnar-Filipowicz A., Hynes N.E. (2016). Decreased NK-cell tumour immunosurveillance consequent to JAK inhibition enhances metastasis in breast cancer models. Nat. Commun..

[37] Wang X., Luo W., Chen Z., Li C., Zhou J., Huang Z., Tang L., Wu J., Wu Z., Li Y. (2025). Co-expression of IL-15 and CCL21 strengthens CAR-NK cells to eliminate tumors in concert with t cells and equips them with PI3k/AKT/mTOR signal signature. J. Immunother. Cancer.

[38] Vogler M., Braun Y., Smith V.M., Westhoff M.K.A., Pereira R.S., Pieper N.M., Anders M., Callens M., Vervliet T., Abbas M. (2025). The BCL2 family: From apoptosis mechanisms to new advances in targeted therapy. Signal Transduct. Target. Ther..

[39] Acharya S., Basar R., Daher M., Rafei H., Li P., Uprety N., Ensley E., Shanley M., Kumar B., Banerjee P.P. (2024). CD28 costimulation augments CAR signaling in NK cells via the LCK/CD3ζ/ZAP70 signaling axis. Cancer Discov..

[40] Yan C., Lu P., Jiang Y., Miao S., Zhao L., Xu X. (2025). 2b4/CD244 signaling in immune regulation and its role in infection, cancer, and immune tolerance. Immunotargets Ther..

[41] Li L., Mohanty V., Dou J., Huang Y., Banerjee P.P., Miao Q., Lohr J.G., Vijaykumar T., Frede J., Knoechel B. (2023). Loss of metabolic fitness drives tumor resistance after car-nk cell therapy and can be overcome by cytokine engineering. Sci. Adv..

[42] Bergamaschi C., Stravokefalou V., Stellas D., Karaliota S., Felber B.K., Pavlakis G.N. (2021). Heterodimeric IL-15 in cancer immunotherapy. Cancers.

[43] Sánchez-Moreno I.S., Lasarte-Cia A., Martín-Otal C., Casares N., Navarro F., Gorraiz M., Sarrión P., Hervas-Stubbs S., Jordana L., Rodriguez-Madoz J.R. (2024). Tethered IL15-IL15rα augments antitumor activity of CD19 CAR-t cells but displays long-term toxicity in an immunocompetent lymphoma mouse model. J. Immunother. Cancer.

[44] Felices M., Lenvik A.J., McElmurry R., Chu S., Hinderlie P., Bendzick L., Geller M.A., Tolar J., Blazar B.R., Miller J.S. (2018). Continuous treatment with il-15 exhausts human nk cells via a metabolic defect. J. Clin. Investig..

[45] Elpek K.G., Rubinstein M.P., Bellemare-Pelletier A., Goldrath A.W., Turley S.J. (2010). Mature natural killer cells with phenotypic and functional alterations accumulate upon sustained stimulation with il-15/il-15rα complexes. Proc. Natl. Acad. Sci. USA.

[46] Zhang Y., Zhuang Q., Wang F., Zhang C., Xu C., Gu A., Zhong W.H., Hu Y., Zhong X. (2022). Co-expression IL-15 receptor alpha with IL-15 reduces toxicity via limiting IL-15 systemic exposure during CAR-t immunotherapy. J. Transl. Med..

[47] Tian G., Courtney A.N., Yu H., Bhar S., Xu X., Barragán G.A., Martinez Amador C., Ghatwai N., Wood M.S., Schady D. (2025). Hyperleukocytosis in a neuroblastoma patient after treatment with natural killer t cells expressing a gd2-specific chimeric antigen receptor and il-15. J. Immunother. Cancer.

[48] Ma L., Zhang K., Xu J., Wang J., Jiang T., Du X., Zhang J., Huang J., Ren F., Liu D. (2024). Building a novel TRUCK by harnessing the endogenous IFN-gamma promoter for cytokine expression. Mol. Ther. J. Am. Soc. Gene Ther..

[49] Liu E., Tong Y., Dotti G., Shaim H., Savoldo B., Mukherjee M., Orange J., Wan X., Lu X., Reynolds A. (2018). Cord blood NK cells engineered to express IL-15 and a CD19-targeted CAR show long-term persistence and potent antitumor activity. Leukemia.

