# Supplementary material for: Constitutive RLI Armoring Enhances CAR-NK Cell Effector Functions but Causes Lethal Toxicity In Vivo
Source: Int J Mol Sci. 2026 Apr 16;27(8):3554. doi: 10.3390/ijms27083554 (PMC13116091; doi:10.3390/ijms27083554)
Supplement: Supplementary file 1 [file ijms-27-03554-s001.zip › ijms-4212200-supplementary.pdf]

Supplementary Table S1. Detailed list of the fluorophore-conjugated antibodies used in flow cytometry experiments.

Marker	Fluorophore	Cat#	Company
CD3	PE	300308	Biolegend
CD56	PE-Cy7	362510	Biolegend
c-myc	APC	626810	Biolegend
CD16	FITC	302006	Biolegend
CD107a	FITC	328606	Biolegend
LAG3	APC	369212	Biolegend
PD-1	APC	329908	Biolegend
CD19	APC	302212	Biolegend
CD45	APC	304012	Biolegend
TIM3	APC	345012	Biolegend
TIGIT	PE	372704	Biolegend

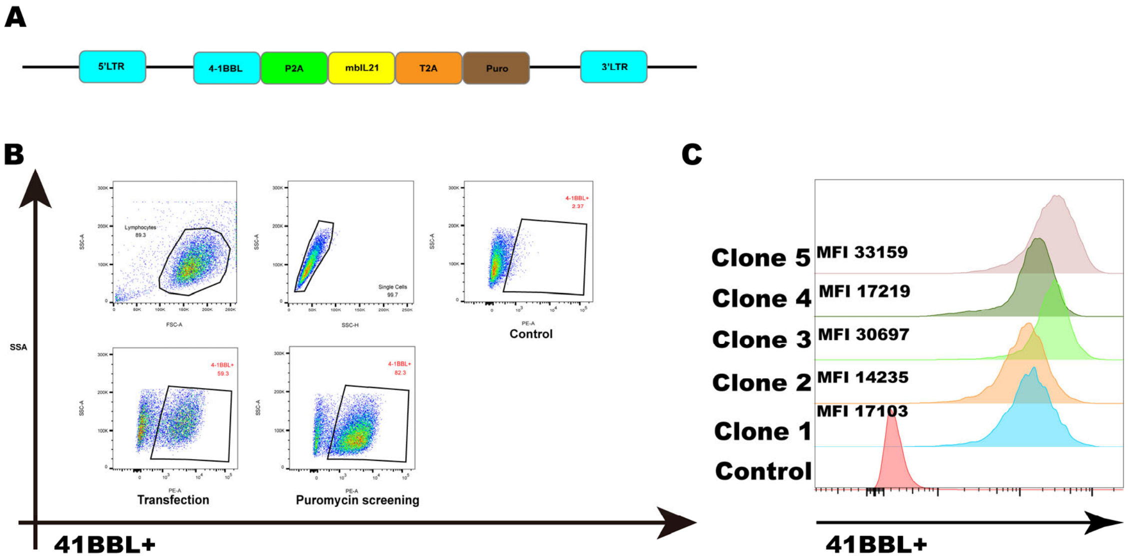

Supplementary Figure S1 Construct of K562 feeder cells. **A** Schematic diagram of K562 feeder constructs. **B** Flow cytometry analysis showing the transfection of K562 feeder cells. **C** The 4-1BBL MFI of the monoclonal K562 feeder cells were measured by flow cytometry.

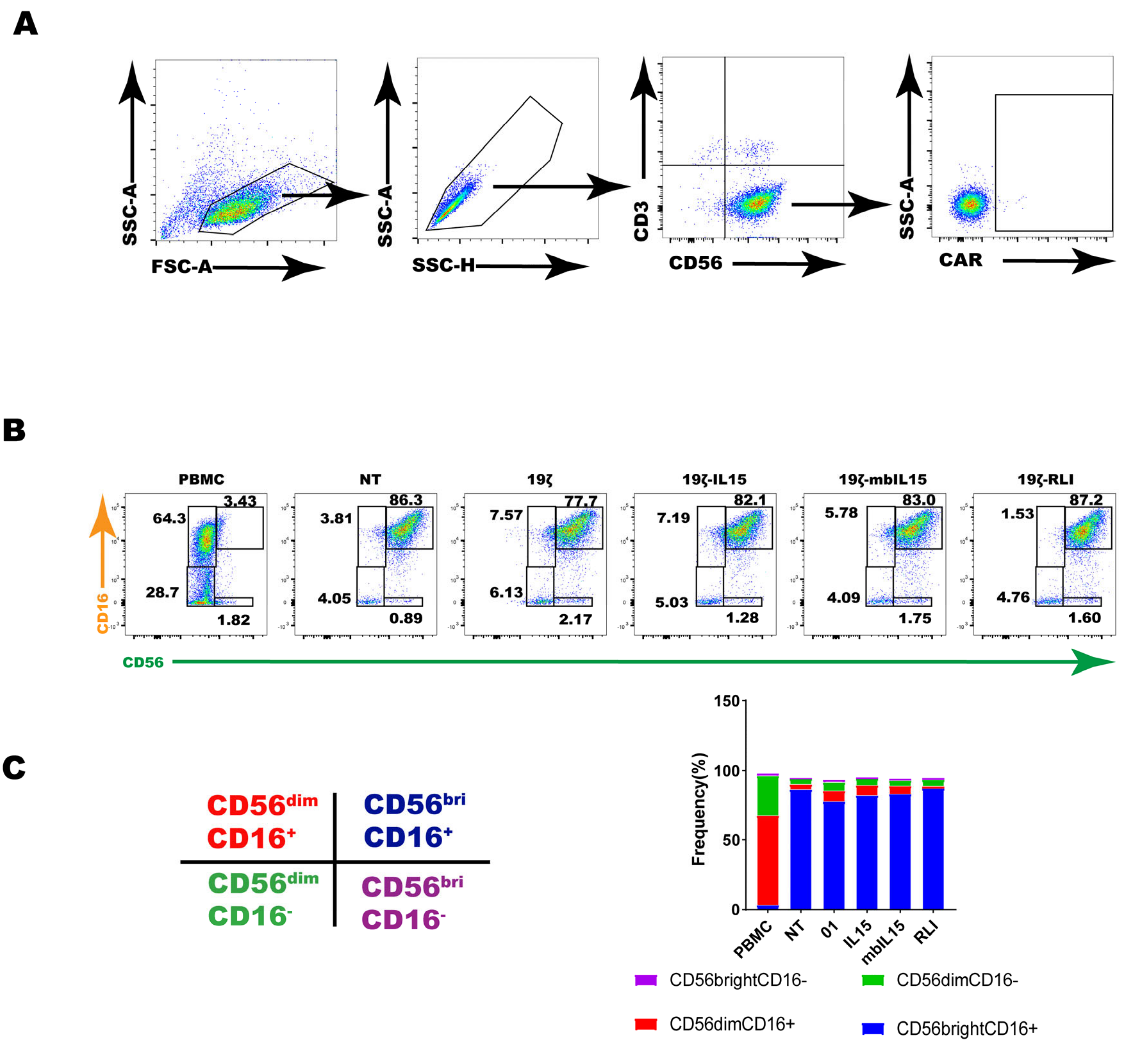

Supplementary Figure S2 Gating strategy of NK cells and subsets. **A** Gating strategy used to identify CAR NK cells. **B-C** Phenotype signatures of CAR-NK cells.

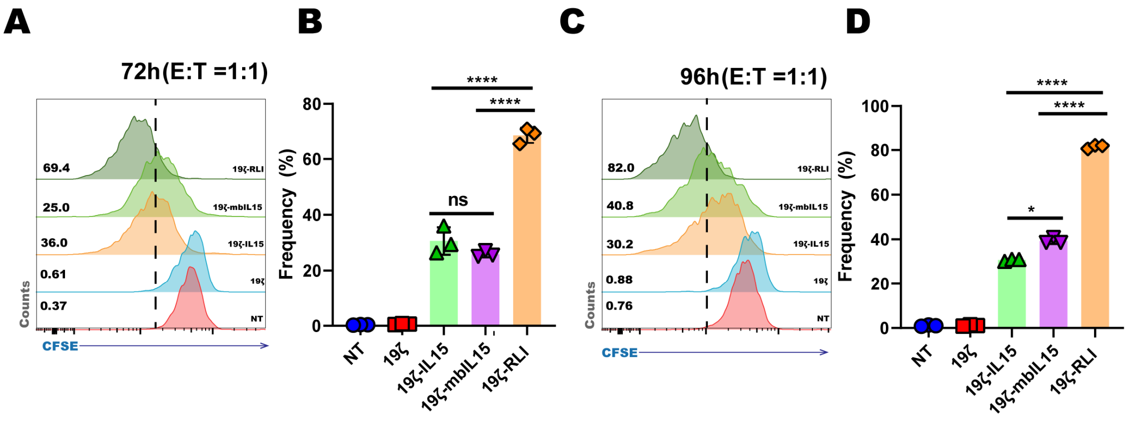

Supplementary Figure S3 CFSE proliferation assay at 72 h and 96 h. CAR-NK cells were co-cultured with Raji cells (E: T = 1:1) for 72 h (**A, B**) or 96 h (**C, D**). Proliferation was measured by CFSE dilution. Representative histograms (**A, C**) and quantification (**B, D**) are shown. Statistical comparisons were performed using one-way ANOVA with Tukey's correction (b, d). Data are presented as the mean ± s.e.m. ns, no significant. *, P<0.05. ****, P<0.0001.

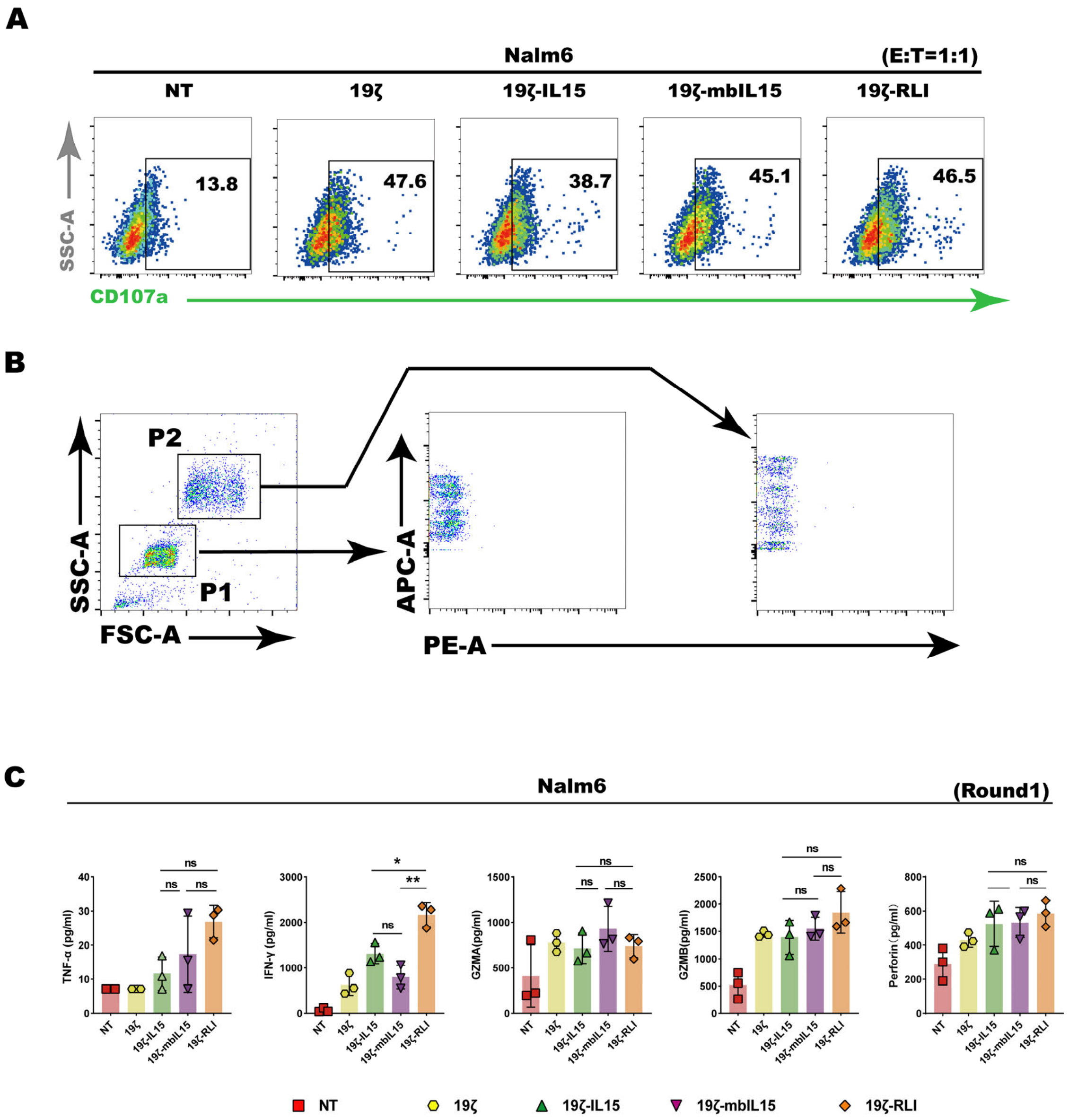

Supplementary Figure S4 Degranulation and cytokine production of CAR NK cells. **A** CAR-NK cells were co-culture with Nalm6 cells at an E:T ratio of 1:1 for 4 h and harvested for analysis of the surface expression of CD107a. FACS plot showed one representative data (n=3). **B** Gating strategy used to identify cytokine production of CAR-NK cells. **C** The supernatant TNFα, IFNγ, perforin, GZMA and GZMB after CAR-NK cells co-culturing with Nalm6 cells for 24hour were measured by ELISA assay(n=3). Statistical comparisons were performed using one-way ANOVA with Tukey's correction (**C**). Data are presented

as the mean ± s.e.m. ns, no significant. *, P<0.05. **, P<0.01.

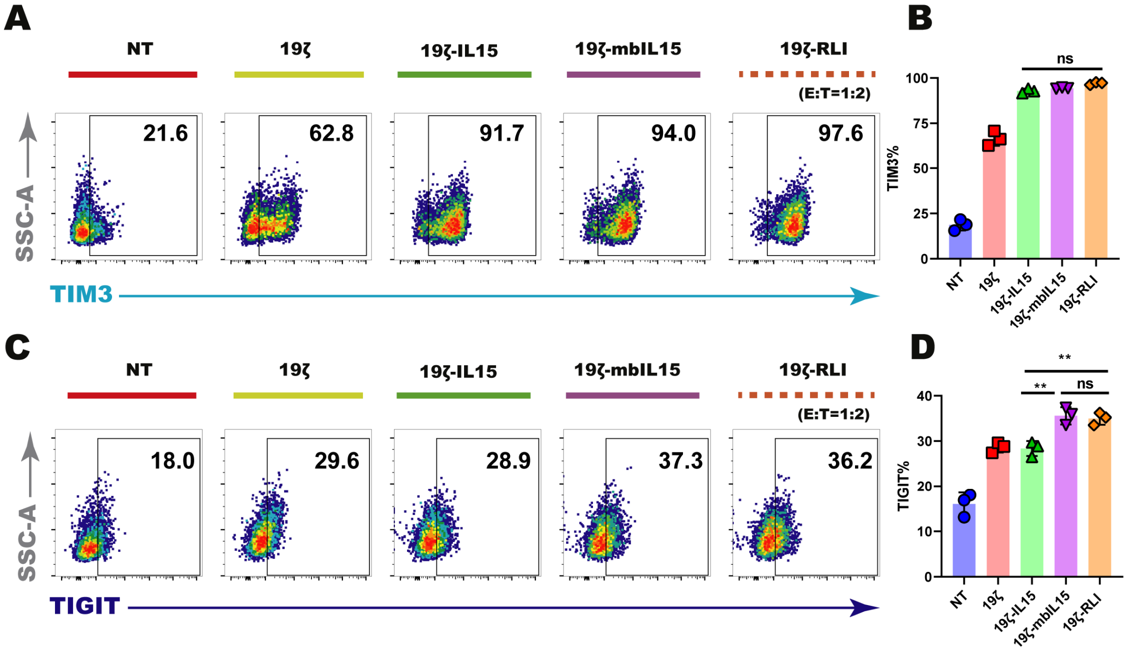

Supplementary Figure S5 TIM-3 and TIGIT expression profiles. Representative flow cytometry plots showing the expression of exhaustion markers (TIM3 and TIGIT) on NT, 19ζ, 19ζ-IL15, 19ζ-mbIL15, and 19ζ-RLI cells after two rounds of tumor rechallenge with Nalm6 cells (n=3). **A, C** Representative flow cytometry plots of TIM-3 (**A**) and TIGIT (**C**) expression. **B, D** Summary data of TIM3 (**B**) and TIGIT (**D**) expression. Statistical comparisons were performed using one-way ANOVA with Tukey's correction (**B, D**). Data are presented as the mean ± s.e.m. ns, no significant. **, P<0.01.

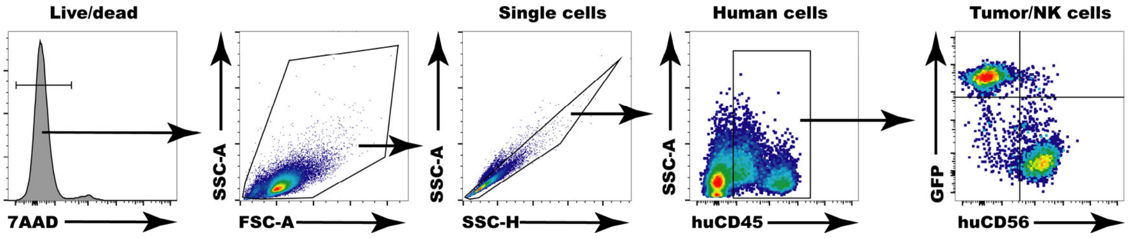

Supplementary Figure S6 Gating strategy used to identify human NK cells and tumor cells *in vivo*.

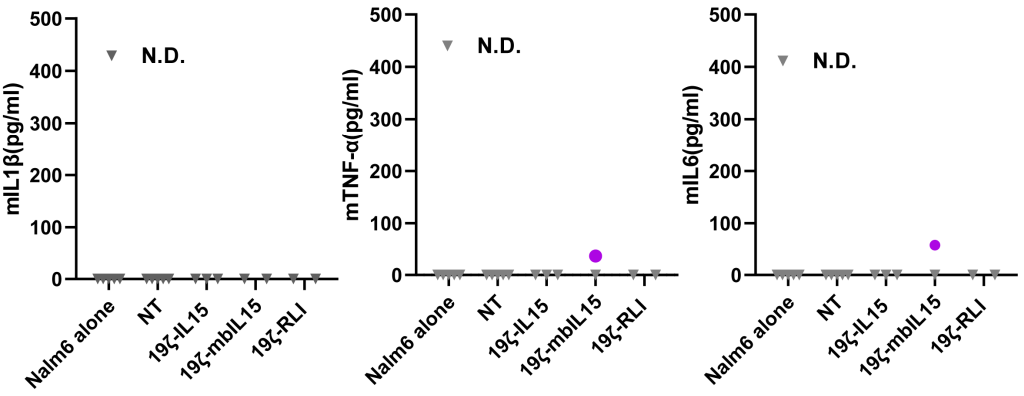

Supplementary Figure S7 Analysis of CRS-associated cytokines. Serum concentrations of mouse IL-1β, TNF-α, and IL-6 were measured by ELISA at the experimental endpoint. Values below the detection limit are shown as zero. N.D., not detectable.

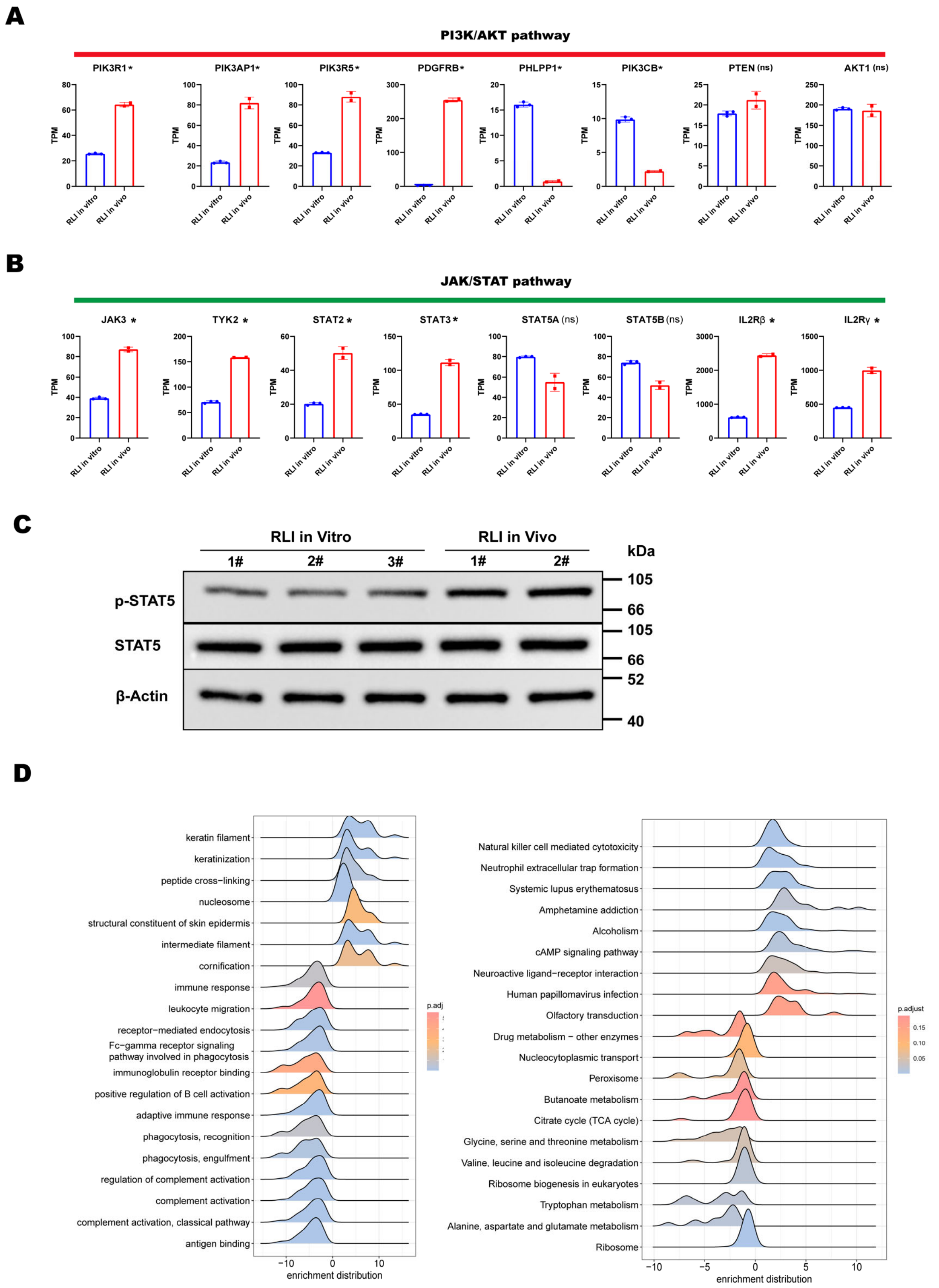

Supplementary Figure S8 Transcriptomic Landscape Underlying Cytokine-Driven Hyperproliferation. **A-B** TPM (transcripts per million) values for genes associated with

PI3K/AKT pathway (**A**) and JAK/STAT pathway (**B**). **C** Phosphorylation levels of STAT5 in in vivo-recovered and in vitro-cultured 19ζ-RLI cell. **D** GSEA Ridgeplot showing enrichment distribution of gene sets.
